# The *Cardamine enshiensis* genome reveals whole genome duplication and insight into selenium hyperaccumulation and tolerance

**DOI:** 10.1038/s41421-021-00286-x

**Published:** 2021-08-10

**Authors:** Chuying Huang, Hongqin Ying, Xibiao Yang, Yuan Gao, Tuo Li, Bo Wu, Meng Ren, Zixiong Zhang, Jun Ding, Jianhua Gao, Dan Wen, Xingzhi Ye, Ling Liu, Huan Wang, Guogen Sun, Yi Zou, Nansheng Chen, Li Wang

**Affiliations:** 1 Hubei Minzu University Affiliated Enshi Clinical Medical School, The Central Hospital of Enshi Tujia and Miao Autonomous Prefecture, Enshi, Hubei China; 2 Hubei Selenium and Human Health Institute, Enshi, Hubei China; 3 Hubei Selenium Industrial Technology Research Institute, Enshi Autonomous Prefecture Academy of Agriculture Sciences, Enshi, Hubei China; 4 Department of Radiology, West China Hospital of Sichuan University, Chengdu, Sichuan China; 5 Department of Chemistry and Molecular Biology, University of Gothenburg, SE 405 30 Gothenburg, Sweden; 6 Center for Bioinformatics and Computational Biology, and the Institute of Biomedical Sciences, School of Life Sciences, East China Normal University, Shanghai, China; 7 South China Potato Research Center, Enshi Autonomous Prefecture Academy of Agricultural Sciences, Enshi, Hubei China; 8 Bureau of Agricultural & Rural Affairs of Enshi Tujia and Miao Autonomous Prefecture, Enshi, Hubei China; 9 Wuhan Frasergen Bioinformatics Co., Ltd., Wuhan, Hubei China; 10 Institute of Oceanology, Chinese Academy of Sciences, Qingdao, Shandong China; 11 Department of Molecular Biology and Biochemistry, Simon Fraser University, Burnaby, Canada

**Keywords:** Plant molecular biology, Plant sciences

## Abstract

*Cardamine enshiensis* is a well-known selenium (Se)-hyperaccumulating plant. Se is an essential trace element associated with many health benefits. Despite its critical importance, genomic information of this species is limited. Here, we report a chromosome-level genome assembly of *C. enshiensis*, which consists of 443.4 Mb in 16 chromosomes with a scaffold N50 of 24 Mb. To elucidate the mechanism of Se tolerance and hyperaccumulation in *C. enshiensis*, we generated and analyzed a dataset encompassing genomes, transcriptomes, and metabolomes. The results reveal that flavonoid, glutathione, and lignin biosynthetic pathways may play important roles in protecting *C. enshiensis* from stress induced by Se. Hi-C analysis of chromatin interaction patterns showed that the chromatin of *C. enshiensis* is partitioned into A and B compartments, and strong interactions between the two telomeres of each chromosome were correlated with histone modifications, epigenetic markers, DNA methylation, and RNA abundance. Se supplementation could affect the 3D chromatin architecture of *C. enshiensis* at the compartment level. Genes with compartment changes after Se treatment were involved in selenocompound metabolism, and genes in regions with topologically associated domain insulation participated in cellular responses to Se, Se binding, and flavonoid biosynthesis. This multiomics research provides molecular insight into the mechanism underlying Se tolerance and hyperaccumulation in *C. enshiensis*.

## Introduction

Selenium (Se) is an essential trace element with antioxidant, anti-inflammatory, and thyroid metabolic regulation properties when incorporated into selenoproteins^1^. Low Se in humans has been associated with increased risk of mortality, poor immune function, cognition decline, and irreversible brain damage^2^. Se supplementation can augment the activity and transcription of glutathione peroxidase 4 (GPX4), effectively inhibiting GPX4-dependent ferroptosis and improving the prognosis of hemorrhagic and ischemic stroke^3^. Recently, Se supplementation has been found to dramatically augment thioredoxin reductase 1 (TXNRD1) activity, which considerably increases anticancer efficiency of cisplatin and TXNRD1 inhibitors (Huang et al., unpublished observations). *Cardamine enshiensis* was initially identified in Se mining areas in Enshi^4^, China, and has since been cultivated at a large scale as a new food source. This plant did not show apparent growth reduction when exposed to 400 µM Se for 3 months, though it accumulated over 3.7% Se by dry weight^5^. Therefore, *C. enshiensis* can be potentially used in, for example, the effective phytoremediation of Se-contaminated soil and water. Indeed, the economic contribution made by Se-related industries accounts for nearly 50% of the annual GDP of Enshi city. Thus, it is both environmentally and economically important to elucidate the mechanism of Se tolerance and hyperaccumulation in *C. enshiensis*.

## Results and discussion

### Genome sequencing, assembly, and annotation

**Fig. 1:**
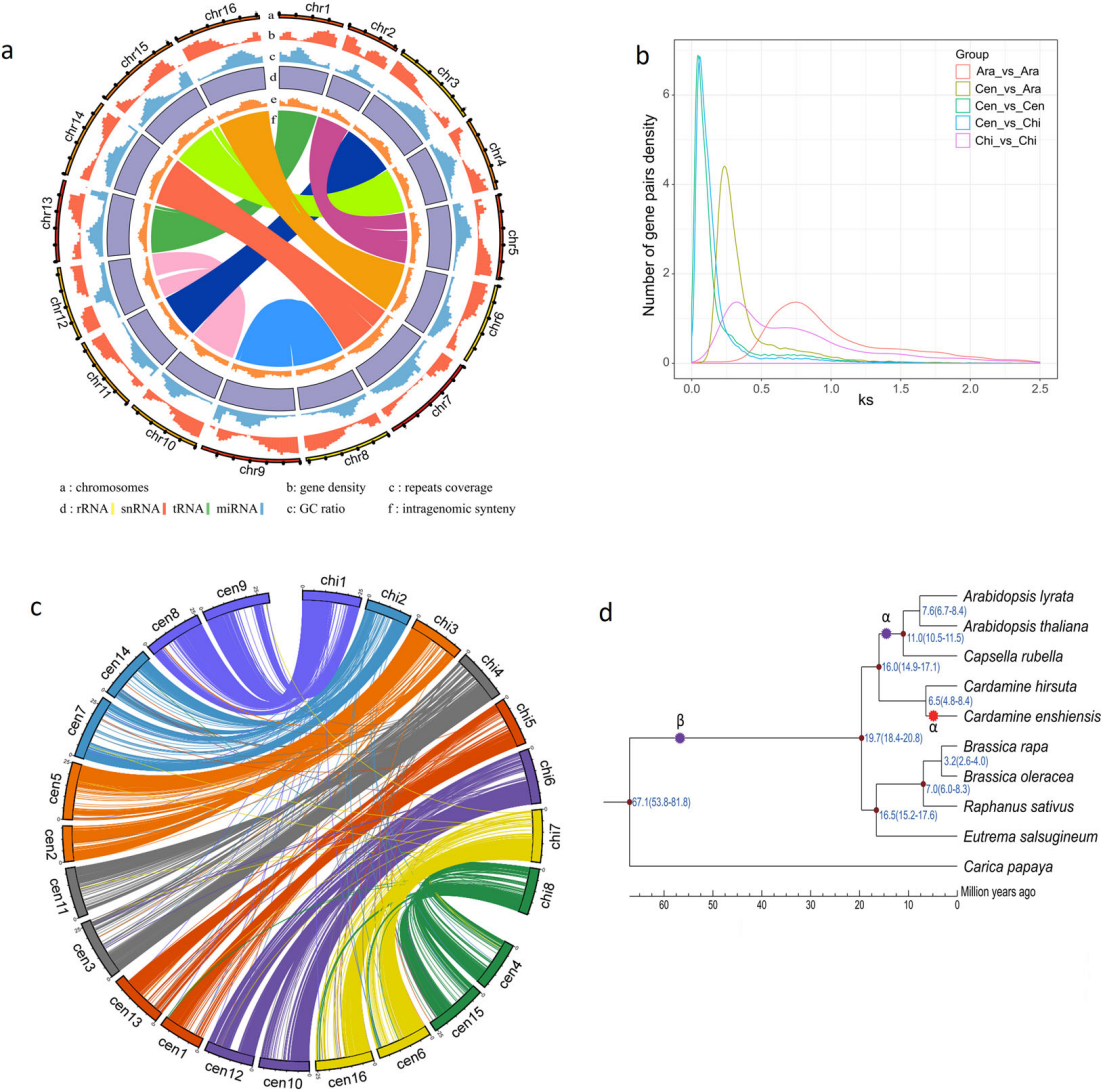
Genomic features and WGD in *C. enshiensis*. **a** Characteristics of the 16 chromosomes of *C. enshiensis* with a window size of 200 kb. The displayed plots are the gene density, repeated density, GC density, RNA, and relationships between syntenic blocks. **b** Synonymous substitution rate (*K*s) distributions of syntenic blocks for *C. hirsuta* paralogs and orthologs. **c** Circos plots showing synteny between the genomes of *C. hirsuta* and *C. enshiensis*. **d** Inferred phylogenetic tree constructed with orthologs across 9 species, including their divergence times and WGDs. The posterior probabilities for all branches exceeded 0.99. α and β: the ancient α and β WGDs occurred about 47 and 124 million years ago.

Here, we sequenced the *C. enshiensis* genome and successfully organized the contigs into 16 pseudochromosomes with 86.6% of the genome represented by the contigs (Fig. 1a). We produced a final genome assembly of *C. enshiensis* with a total length of 443.46 Mb (2*n* = 32), a contig N50 of 1.23 Mb and a scaffold N50 of 24.41 Mb (Table 1; Supplementary Table S1). The genome size of *C. enshiensis* was close to that estimated by Kmer analysis (481.37 Mb) (Supplementary Fig. S1). Benchmarking Universal Single-Copy Orthologs (BUSCO)^6^ analysis demonstrated that 97.7% of the genes were identified^7^ (Supplementary Table S2). We annotated the genome using the Maker pipeline^8^, incorporating protein homolog, de novo prediction, and transcriptome data prediction. These results were integrated into a final set of 52,725 gene models with an average length of 2.1 kb, an average coding sequence length of 1.1 kb, and an average of 5.1 exons per gene (Supplementary Table S3). The majority of the predicted genes (96.0%) were supported by homology and were functionally annotated, with 76.3% supported by the InterPro database^9^ (Supplementary Table S4). Using InterProScan, we annotated 27,391 genes on the basis of Gene Ontology (GO) classification. This annotation also predicted 3324 noncoding RNAs (ncRNAs) (Supplementary Table S5). Repetitive elements comprised 61.4% of the genome, and 48.5% were long terminal repeat (LTR) retrotransposons (Supplementary Tables S6–S8).

**Table 1:** Statistics of assembled genome of *C. enshiensis*.

Assembly feature	Statistic
Estimated genome size (by k-mer analysis)	481.37 Mb
Number of scaffolds	2267
Number of cotigs	3289
Scaffold N50	24.41 Mb
Cotig N50	1.23 Mb
Longest cotig	9.63 Mb
Longest scaffold	29.97 Mb
Assembly length	443.46 Mb
Assembly % of genome	97.7
GC content	36.27%
Repeat density	61.35%
Predicted gene models	52,725

### Evolution of the *C. enshiensis* genome and comparative genomic analysis

Collinearity analysis uncovered a whole genome duplication (WGD) event and segmental duplication after WGD (Fig. 1b; Supplementary Fig. S2 and Table S9). KEGG analysis revealed that ferroptosis was the most highly enriched term (Supplementary Table S10). Phylogenetic analysis revealed that the divergence time between *Cardamine* and *Arabidopsis thaliana* was ~16 MYA, while that between *C. enshiensis* and *Cardamine hirsuta* (2*n* = 16)^10^ was ~6.5 MYA (Fig. 1d). Using the *K*s value and divergence times, we calculated the rate of synonymous substitution per site per year to be 8 × 10^–9^, corresponding to the WGD event, which occurred around 5 MYA (Fig. 1b, d; Supplementary Fig. S2). A CIRCOS plot showed a clear 2:1 syntenic relationship between *C. enshiensis* and *C. hirsuta*, and each chromosome of *C. hirsuta* was highly collinear with the corresponding chromosome of *C. enshiensis* (Fig. 1c), suggesting that *C. enshiensis* is tetrapolidy.

### Effects of Se on the 3D genome architecture of *C. enshiensis*

**Fig. 2:**
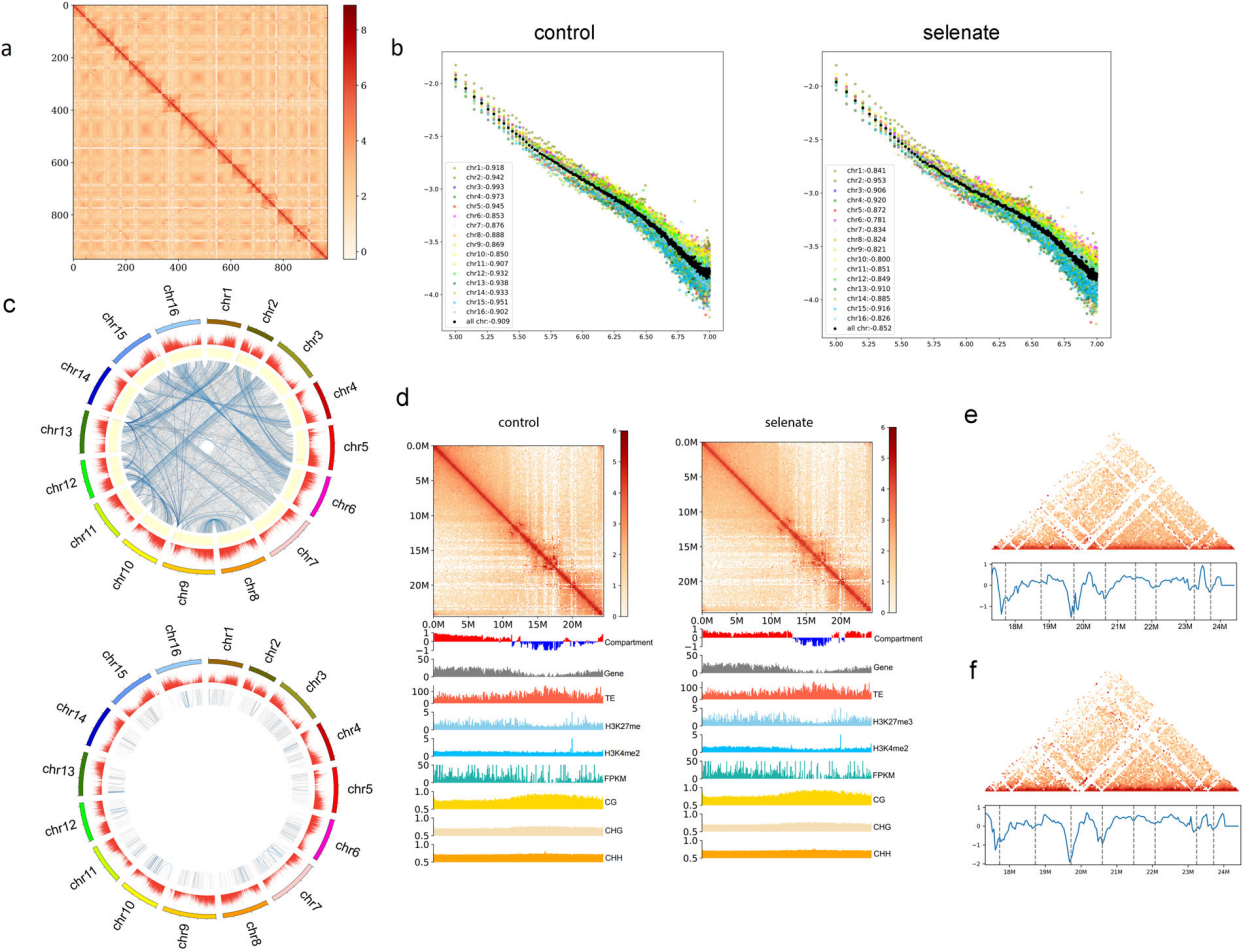
Hi-C contact maps and genome-wide contact matrix of *C. enshiensis*. **a** Hi-C interactome (400-kb bins) within and among *C. enshiensis* chromosomes (Chr1–Chr16). Intrachromosomal interactions were observed between the euchromatin arms of all chromosomes. The color intensity represents the frequency of contact between two 400-kb loci. Boxes along the diagonal area: interactions within the same chromosome (*cis*). The white rows and columns indicate bins with no valid interaction data. **b** Whole-genome distance-interaction frequency diagram with a 400-kb resolution between the samples. IDE values within each chromosome with or without Se supplement. Color: interaction attenuation curve of different samples; horizontal axis: relative distance between different sites on the chromosome; vertical axis: interaction frequency. **c** Circos plots showing genome-wide significant *cis*-interaction sites (upper) and *trans*-interaction sites (lower). **d** Hi-C interaction map and colocalization of the genomic composition and various epigenetic marks on Chr6. The heatmap denotes the intrachromosomal Hi-C interaction frequencies among the pairwise 100-kb bins shown on the top. The PCA eigenvectors of the A and B compartments and genomic and epigenetic feature tracks are shown below in separate 100-kb bins, including abundances of genes and TEs, different histone modifications, mRNA expression levels (normalized by FPKM), and DNA methylation (in CG, CHG, and CHH contexts). **e**, **f** Hi-C interaction matrix of a region (**e** chr6.116240000-24160000; **f** chr6.17280000-24840000) showing the TADs. Top, Hi-C interaction matrix; bottom, TAD boundaries (vertical bars) and insulation scores. The vertical axis and the blue line in the figure represent the insulation scores, and the gray line in the figure shows the TAD boundary.

To investigate the effects of Se on the 3D genome architecture of *C. enshiensis*, we performed Hi-C experiments on *C. enshiensis* before and after Se treatment. Hi-C experiments have been widely used to study the genomes of bacteria, yeast, *A. thaliana*, cotton, rice, maize, mouse, and human^11–20^. In this study, using the HiSeq platform and 150-bp paired-end (150PE) mode, a total of 23 million valid paired reads were generated for comparative 3D genome analysis. We also performed DNA sequencing, DNA methylation sequencing, RNA sequencing, and chromatin immunoprecipitation and subsequent sequencing (ChIP-seq) for two histone modifications using the same leaf tissue samples. After normalizing the data with Iterative Correction and Eigenvector (ICE) software^21^, we constructed 400-kb genome-wide Hi-C interaction maps and 100-kb chromosome-wide Hi-C interaction maps (Fig. 2a, d). Collinearity analysis of the genome of *C. enshiensis* yielded 8 pairs of putative homoeologous chromosomes (Fig. 1a). Interactions between putative homoeologs accounted for 10.63%–27.29% of all chromosome interactions (Supplementary Table S11). These results revealed that the predominant telomere interactions of *C. enshiensis* occurred among all telomeres of all chromosomes, and this result was comparable to that in *A. thaliana*^12^. We also detected relatively independent and strong intrachromosomal interactions in the pericentromeric areas and interchromosomal interactions. We then used the 3D genome maps to identify 25,000 pairs of significant interaction sites, including 1186 *cis*-interactions and 7050 *trans*-interactions in the control group and 774 *cis*-interactions and 7345 *trans*-interactions in the selenate treatment group (Fig. 2c). To better and more intuitively show the patterns of chromatin interaction between pairs of samples, we used the method by Crane et al.^22^ to transform the interaction matrix of each sample into a z-score matrix and then subtracted the z-score matrix of the two samples to obtain a subtractive interaction matrix (Supplementary Fig. S3b). Distance-dependent interaction decay, measured by interaction decay exponents (IDEs)^23,24^, has been used to describe trends in interaction frequency with distance. To reveal whether selenate affects spatial structure of the *C. enshiensis* chromosomes, we combined the two contact decay curves to observe the overall variation in the trends of genome-wide interaction decay between samples^15^. There was a large difference between the two groups (Fig. 2b; Supplementary Fig. S3a, Table S12). We found that the genome-wide IDEs of both groups (IDEcontrol: –0.908, IDEse: –0.8518) were consistent with the IDEs of *A. thaliana*, rice, and metazoan species^24–26^. The A and B compartments were identified by genome-wide eigenvector analysis^14^, and similar organization was also observed in *A. thaliana*^24^, maize, tomato^13^, and rice^26^. We used the C-score^27^ to investigate the genomic compartments of *C. enshiensis*, and conspicuous A and B compartments were observed (Fig. 2a). The A compartment displayed a higher gene density, active epigenetic markers (H3K4me2), and high transcription activity, while the B compartment had a lower proportion of repressive epigenetic markers (H3K27me3), more methylated cytosines, and a greater transposable element (TE) density (Supplementary Fig. S3d). Intriguingly, selenate remodels the A and B compartments of chromosomes 2, 6, 8, 9, and 10 (Fig. 2d; Supplementary Fig. S4). To understand the effect of selenate on these compartments, we analyzed the compartments of the selenate-treated and control samples at 100-kb resolution, and 755 compartment differences were identified between the two groups. The conserved A compartment domain had a higher gene density and activated epigenetic markers, while the conserved B compartment domains and differential compartment domains were associated with an increased TE density and decreased repressive epigenetic markers (Supplementary Fig. S5). To explore the functions of genes located on chromatin with compartment differences, the gene sets in each compartment were functionally annotated, and separate GO and KEGG analyses were conducted (Supplementary Figs. S6 and S7). The significantly enriched pathways were concentrated in glyoxylate and dicarboxylate metabolism, fat digestion and absorption, insert hormone biosynthesis, and selenocompound metabolism.

Topologically associated domains (TADs) are basic organizational units of spatial genomic structure. These domains are pervasive in the mammalian genome^28^, and many TAD-like domains have also been identified in plants. TAD boundaries are rich in promoter-related transcription factors, transcription initiation sites, housekeeping genes, tRNA genes, and short interspersed nuclear elements (SINEs), which play an important role in maintaining the structure and stability of TADs^28^. The boundaries of TAD structures in mammalian genomes tend to be enriched in CTCF binding sites^29^, while multiple histone modification signals are enriched in plant genomes, the chromatin is more open^17^, and the genes show higher expression levels^20^. TADs were identified in *C. enshiensis* using Hi-C interaction maps at 40-kb resolution (Fig. 2e, f). We identified 537 and 543 TADs and 521 and 527 TAD boundaries in the control group and 400 µM sodium selenate treatment group, respectively (Supplementary Table S13). Intriguingly, we found that TAD boundaries had higher gene densities and gene expression levels compared to TAD interior regions (Supplementary Fig. S8). To examine the changes in genome methylation sites at the TAD level, we used a sliding window method to analyze the differential insulation between the two groups^22^. TAD boundaries had higher active epigenetic marks (H3K4me2) and more methylated cytosines in CHG contexts compared to TAD interior regions, respectively (Supplementary Fig. S8). We examined the gene expression data and found that genes were enriched in cellular response to Se, Se binding, and flavonoid biosynthesis. Gene annotation and GO and KEGG analyses were conducted to investigate the function of genes in the changed boundaries. The significantly enriched pathways of the genes in the selenate treatment group gained boundaries were concentrated in the cellular response to Se and Se binding (Supplementary Fig. S9). We also used sliding window (window size: 11, step size 1) method to analyze regions with different insulation scores (Pearson correlations score < 0.6 in all the overlapped windows) between the two groups^22^. The significantly enriched pathways among these regions were concentrated in the flavonoid biosynthesis (Supplementary Fig. S10). Overall, these results suggest that Se tolerance and metabolism have correlations at the chromatin level. Furthermore, selenate remodels the TADs of the *metE*, *GS*, and *PAL* genes (Figs. 4d and 5c; Supplementary Fig. S11d).

Frequently interacting regions (FIREs) are hotspots of local chromatin interactions that are distinct from compartments, TADs, and now loops^22^. FIREs indicate significantly high-frequency local chromatin interactions and have been demonstrated to be enhancer enrichment regions with a higher probability of superenhancers^30^. In total, 1184 significant FIREs were identified at a 10-kb resolution, and these regions were remarkably enriched on chromosomes 2, 9, and 13, in compartment A, and in TAD boundary regions (Supplementary Figs. S3c and S12). We have examined the gene density distribution and GC content of FIRE concentration sites, and found that there is no significant difference compared to other regions. Hence, these regions are not small gene island regions separated by inactive and condensed heterochromatin (Supplementary Fig. S13).

### Mechanisms of Se tolerance and hyperaccumulation in *C. enshiensis*

**Fig. 3:**
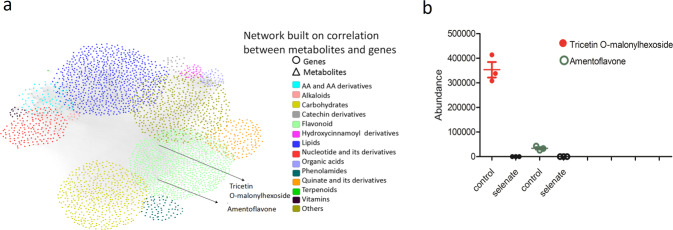
Flavonoids enhance the tolerance of *C. enshiensis* to Se. **a** The connection network between 29,671 differentially expressed regulatory genes and 127 differential metabolites. The networks were visualized through Cytoscape software. **b** *C. enshiensis* seedlings were treated with 400 µM sodium selenate or water as a control for 24 h. The levels of tricetin O-malonylhexoside and amentoflavone were measured by LC–MS/MS.

To better understand the mechanisms of Se tolerance in *C. enshiensis*, we treated seedlings with 400 µM sodium selenate or water as a control for 24 h. In total, 29,671 differentially expressed genes were identified, accounting for 66.6% of the annotated genes (Supplementary Table S14). To further investigate the variation in the metabolome after Se treatment, we selected leaves from the two groups for metabolite quantification. A broadly targeted liquid chromatography–tandem mass spectrometry (LC–MS/MS)-based metabolic profiling method was used to quantify the metabolites. A total of 558 metabolites were identified in the leaf tissue of *C. enshiensis* (Supplementary Fig. S14, Table S15), including 127 differential metabolites. A high concentration of Se can generate oxidative stress through the overproduction and accumulation of reactive oxygen species (ROS) and reactive nitrogen species (RNS) in plants^31^. Flavonoids enhance the tolerance of plants to heavy metals by acting as metal chelators and scavenging ROS^32^. Intriguingly, we found that 10 flavone-related metabolites were altered between the two groups, indicating that flavones play a pivotal role in Se tolerance (Supplementary Table S15). KEGG analyses of the different metabolites were conducted, and the significantly enriched pathways were primarily associated with the biosynthesis of secondary metabolites and flavone/flavonoid/flavonol compounds (Supplementary Fig. S15). The levels of tricetin O-malonylhexoside and amentoflavone became undetectable, whereas that of luteolin O-hexosyl-O-hexosyl-O-hexoside was increased by 24,444-fold (Fig. 3b; Supplementary Table S15). A principal component analysis (PCA) of all the metabolite data indicated transcriptome and metabolome differences between the two groups (Supplementary Fig. S16). To understand the patterns linking the transcriptome and metabolome, correlation analyses were carried out using the data from the two groups. A Pearson’s correlation coefficient threshold of *r* > 0.8 was used to identify the metabolites that were significantly correlated with each gene. In total, 105,268 expression correlations involving 183 metabolites and 3202 genes were identified (Fig. 3a). Next, we integrated the above data by building a network to facilitate metabolic pathway and candidate gene identification. In total, 2000 transcripts and 2 flavonoid metabolites were subjected to Pearson’s correlation analysis, and the results revealed that 175 transcripts were highly correlated (*R*^2^ > 0.96) with tricetin O-malonylhexoside and amentoflavone (Supplementary Table S16).

**Fig. 4:**
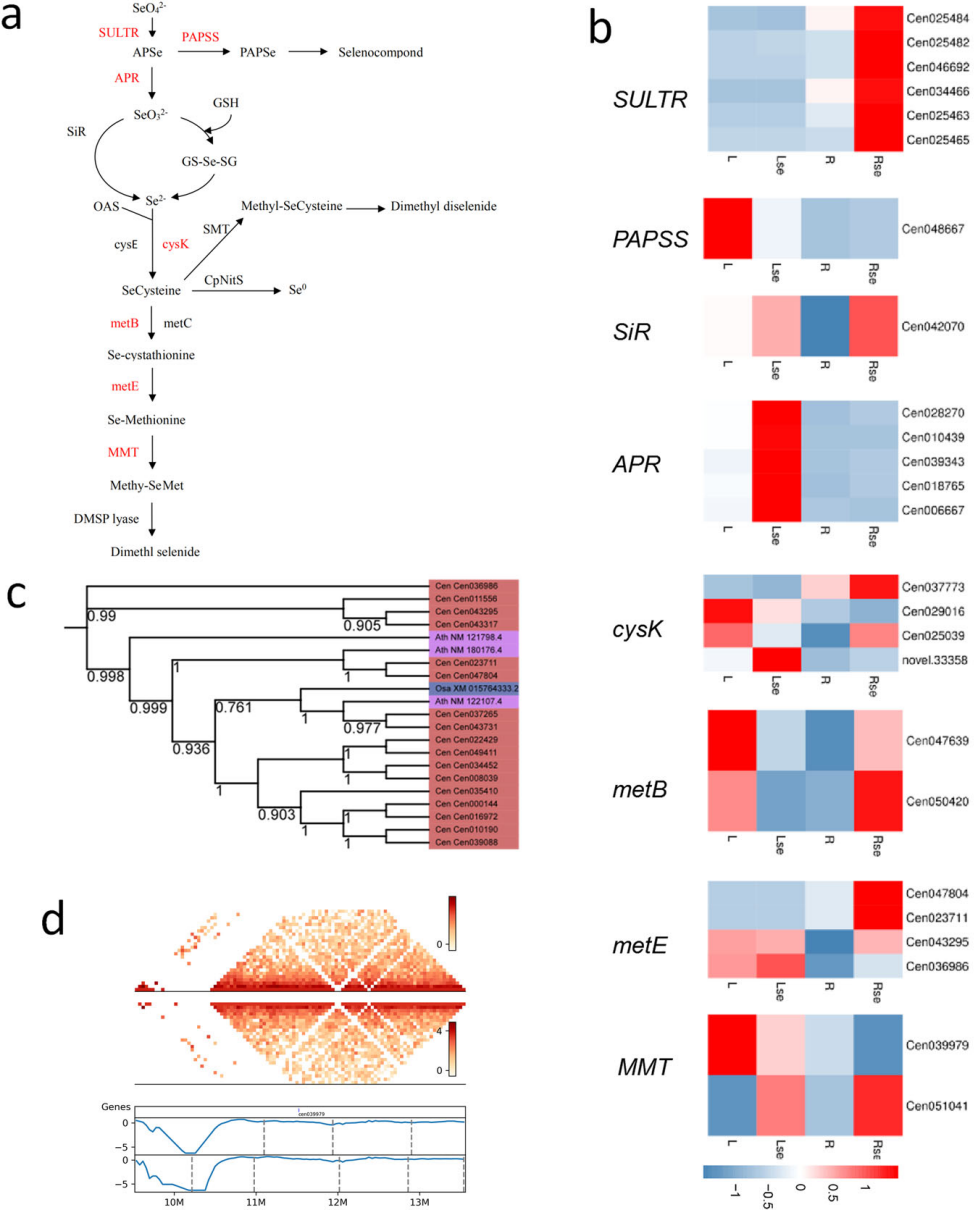
Se metabolism in *C. enshiensis*. **a** The reported pathway and genes that regulate Se metabolism. The red-labeled genes are identified in *C. enshiensis*. **b** Tissue-specific expression of Se metabolic pathway genes across different tissues. L, leaf tissue from control group; Lse, leaf tissue from Se supplement group; R, root tissue from control group; Rse, root tissue from Se supplement group. **c** Phylogenetic tree of the *metE* gene family across *C. enshiensis* and other plants. **d** Hi-C interaction matrix (cen039979, chr4, 11482047–11488608, 2 Mb range upstream and downstream of the *MMT* gene) shows interaction and TAD signal (40-kb resolution). Top, Hi-C interaction matrix; bottom, TAD boundaries (vertical bars) and insulation scores. The vertical axis and the blue line in the figure represent the insulation score, and the gray line in the figure shows the TAD boundary.

**Fig. 5:**
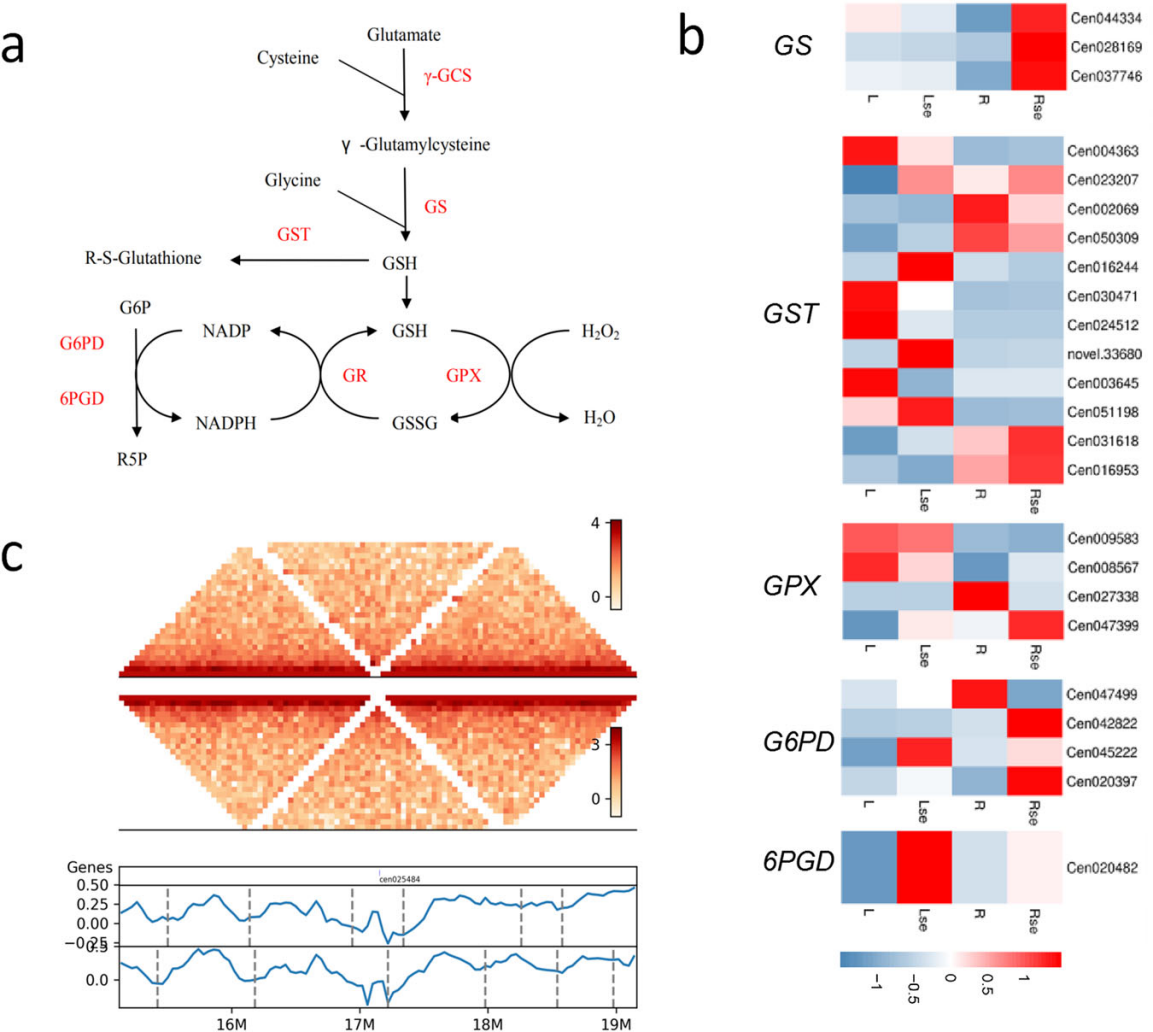
GSH is associated with Se tolerance. **a** The reported genes in the GSH metabolic pathway. The red-labeled genes are identified in *C. enshiensis*. **b** Tissue-specific expression of GSH metabolic pathway genes across different tissues. L, leaf tissue from control group; Lse, leaf tissue from Se supplement group; R, root tissue from control group; Rse, root tissue from Se supplement group. **c** The Hi-C interaction matrix (cen025484, chr13, 17113723-17116459, 2 Mb range upstream and downstream of the *GS* gene) shows interactions and TAD signals (40-kb resolution). Top, Hi-C interaction matrix; bottom, TAD boundaries (vertical bars) and insulation scores. The vertical axis and the blue line in the figure represent the insulation score, and the gray line in the figure shows the TAD boundary.

To investigate the mechanism of Se hyperaccumulation, we treated *C. enshiensis* seedlings with 400 µM sodium selenate for two weeks and then performed RNA sequencing (Supplementary Tables S17 and S18). Our genomic and transcriptomic analyses identified 8 genes in the Se metabolic pathway: sulfate transporter (*SULTR*), 3′-phosphoadenosine 5′-phosphosulfate synthase (*PAPSS*), adenylylsulfate reductase (*APR*), sulfite reductase (*SiR*), cysteine synthase (*cysK*), cystathionine gamma-synthase (*metB*), methionine synthase (*MetE*), and met S-methyltransferase (*MMT*) (Fig. 4a). The expression of these genes changed after Se treatment in *C. enshiensis*. The *SULTR*, *SiR*, *cysK*, *metB*, *MetE*, *MMT* genes in root tissue and *SiR*, *APR*, *MetE* genes in leaf tissue were upregulated (Fig. 4b). Se accumulation and volatilization are particularly attractive for the phytoremediation of Se-contaminated environments because inorganic Se is converted to the gas dimethylselenide (DMSe)^33,34^. DMSe is the major volatile Se produced by plants and is ~600 times less toxic than inorganic Se^35^. MMT was shown to be a rate-limiting enzyme in the enzymatic pathway involved in Se volatilization. Mutating this gene resulted in an almost complete loss of the capacity for Se volatilization in *Arabidopsis*^36^. Among the 8 genes identified in the Se metabolic pathway, the *MMT* gene showed the highest expression (Supplementary Table S19). Its WGD-derived duplicates are homologous to the expanded *metE* gene family associated with Se tolerance (Fig. 4c). Glutathione (GSH) is a key component in metal scavenging due to the high affinity of metals for its thiol (-SH) group and as a precursor of phytochelatins^37^. GSH also plays an important role in protecting plants from the oxidative stress induced by heavy metal exposure^38^. Using transcriptome analysis, 5 genes encoding enzymes in the GSH metabolic pathway were identified (Fig. 5a, b). Glucose-6-phosphate dehydrogenase (G6PD) is a cytosolic enzyme involved in producing nicotinamide adenine dinucleotide phosphate (NADPH), which is responsible for the reduction of glutathione disulfide (GSSG) to GSH^39^. Se treatment strongly upregulated the expression of G6PD (8.5-fold increase relative to the control), suggesting that Se stress resulted in excessive production of NADPH. The expression of GSH synthetase (GS) was also upregulated.

Cell walls are considered to be an important site of metal ion storage and play significant roles in heavy metal hyperaccumulation and hypertolerance^40,41^. Interestingly, significant genomic expansions related to cell wall metabolism, such as lignin, pectin, cellulose, and glucan biosynthesis, were observed in *C. enshiensis* (Supplementary Table S20). Lignin may play an important role in cadmium tolerance and accumulation^42^. In addition, comparative genomics analysis revealed that the lignin biosynthesis pathway was overrepresented, and 30 related genes were identified, including *PAL*, *4CL*, *CCR*, *CAD*, *CCOAOMT*, *F5H*, *COMT*, and *POX* (Supplementary Tables S19 and S21, Fig. S11a, b). Phenylalanine ammonia lyase (PAL) is a key enzyme in the phenylpropanoid pathway that catalyzes the deamination of phenylalanine to *trans*-cinnamic acid, a precursor for the lignin and flavonoid biosynthesis pathways^43^. We observed significant genomic expansion of *PAL*, and a phylogenetic analysis of *PAL* showed that the *PAL* gene duplicates *PAL1* and *PAL2* were the results of a *C. enshiensis*-specific WGD (Supplementary Fig. S11c). Intriguingly, KEGG analysis suggests that ferroptosis, MAPK signaling pathway, phenylalanine biosynthesis, selenocompound metabolism, sulfur metabolism, and flavonoid biosynthesis are the prominent pathway enriched in duplicated genes of chromosome 13 from the WGD (Supplementary Table S22).

DNA methylation and chromatin remodeling are involved in regulating gene expression in response to abiotic stresses^44^. A genome-wide investigation of DNA methylation in the leaves was performed on the 14th day of sodium selenate treatment using the Illumina sequencing platform and 150PE mode. The results showed that the amount of DNA methylation was affected by Se stress, the average methylation level on chromosome 6 was lower in the control group compared with Se stress group (0.6066 vs 0.6803) (Supplementary Fig. S17).

In conclusion, this study revealed a WGD event specific to *C. enshiensis* and provided insights into its evolution. This multi-omic research also provided insights into the mechanisms of Se tolerance and hyperaccumulation in *C. enshiensis*.

## Materials and methods

### Growth and treatment

*C. enshiensis* was obtained from Hubei Se Industrial Technology Research Institute, Enshi in Southwest China. C. *enshiensis* was grown in green house under natural light with daily temperatures ranging from 20 °C to 30 °C. One-month-old leaves were used for DNA extraction. Two-month-old plants were treated with 400 µM sodium selenate or water as a control for 24 h; leaves and roots were used for RNA extraction and metabolite quantification. Two-month-old plants were treated with 400 µM sodium selenate or water as a control for 2 weeks; leaves and roots were used for DNA and RNA extraction, and further transcriptome sequencing and ChIP-seq.

### DNA extraction and sequencing

For DNA extraction, fresh and healthy leaves were harvested from the best-growing *C. enshiensis* individual and immediately frozen in liquid nitrogen, followed by preservation at −80 °C in the laboratory prior to DNA extraction. 50 μg of high-quality genomic DNA was extracted from leaves using a modified CTAB method^45^. RNase A was used to remove RNA contaminants. The quality and quantity of the extracted DNA were examined using a NanoDrop 2000 spectrophotometer (NanoDrop Technologies, Wilmington, DE, USA) and electrophoresis on a 0.8% agarose gel, respectively. A single band corresponding to high molecular weight was observed, and we use Femto Pulse to further verify the DNA size longer than 30 kb, indicating high integrity of DNA molecules for library construction for the Illumina HiSeq X Ten (Illumina Inc., San Diego, CA, USA) and the PacBio Sequel (Pacific Biosciences of California, Menlo Park, CA, USA) sequencing platforms. Using the DNA preparation, a library with an insert size of 350 bp was constructed for the Illumina HiSeq X Ten sequencing platform according to the manufacturer’s protocol. 29.9 Gb short reads were obtained. The HTQC package was used to filter out low-quality bases and reads. Adapter sequences and reads with > 10% N bases or > 50% low-quality bases (≤ 5) were eliminated. Finally, we obtained 24.8 Gb (~56×) of cleaned data for the following genome survey analysis and for final-stage base-level genome sequence correction.

A SMRTbell library with a 20-kb insert size was constructed with BluePippin size selection (Sage Science). The resulting SMRTbell templates were sequenced on eight SMRT cells of the PacBio Sequel platform (Pacific Biosciences, Frasergen, Wuhan, China), generating 8.03 million subreads with a total length of 61.38 Gb for genome assembly.

### Iso-Seq analysis of mRNAs

We analyzed the full-length transcripts of *C. enshiensis* using the Iso-Seq protocol^46^. Total RNA was extracted from a sample containing the combined stem, root, and leaf tissues of the same *C. enshiensis* individual used for genome sequencing with TRIzol reagent (Thermo Fisher Scientific, Waltham, MA, USA; Cat# 15596018). Four SMRT cell libraries were constructed with BluePippin size selection of 1–3 kb and > 3 kb insert sizes (Sage Science, MA, USA) and sequenced on the PacBio Sequel platform, yielding 42.654 Gb of subreads. In addition, a transcriptome library for RNA sequencing was constructed according to the Illumina TruSeq RNA library protocol, and the Illumina HiSeq Platform was used for transcriptome sequencing.

### Transcriptome and metabolite profiling

RNA was extracted from the roots and leaves of *C*. *enshiensis* using TRIzol reagent according to the manufacturer’s instructions. Transcriptome libraries were produced and sequenced on the Illumina HiSeq 2500/×. We filtered out the low-quality reads by following the quality control procedures used for the genome assembly. The transcriptome assembly for *C. enshiensis* was generated using Trinity^47^ with the default parameters. The gene expression levels were computed as the number of reads per kilobase of gene length per million mapped reads (FPKM) using RSEM software^48^. Metabolite profiling was carried out using a widely targeted metabolomic method by Wuhan Metware Biotechnology Co., Ltd. (Wuhan, China) (http://www.metware.cn/). A liquid chromatography–electrospray ionization–tandem mass spectrometry (LC–ESI–MS/MS) system was used for relative metabolite quantification. Cytoscape software was used to build a network of genes and metabolites.

### ChIP-seq

*C. enshiensis* samples (3 g) were washed twice in cold PBS, crosslinked with 1% formaldehyde for 10 min at room temperature, and then quenched by the addition of glycine (125 mmol/L final concentration). Afterwards, the samples were lysed, and chromatin was obtained on ice. The chromatin samples were sonicated to obtain soluble sheared chromatin (average DNA length of 200–500 bp). Twenty microliters of chromatin was saved at –20 °C as input DNA, and 100 µL of chromatin was used for immunoprecipitation with H3K27me3 antibodies (CST9733, Cell Signaling Technology) and H3K4me2 antibodies (CST9725, Cell Signaling Technology). The immunoprecipitated DNA was used to construct sequencing libraries following the protocol provided by the I NEXTFLEX^®^ ChIP-Seq Library Prep Kit for Illumina^®^ Sequencing (NOVA-514120, Bioo Scientific) and sequenced on Illumina X Ten with the 150PE method by Wuhan IGENEBOOK Biotechnology Co., Ltd (http://www.igenebook.com).

### Data analysis

The clean reads were then mapped to the reference genome by BSseeker software^49^. CGmapTools software was used to determine the depth of sequencing of C bases across the genome^50^. The methylation level was determined by dividing the number of reads covering each methylated C site (mC) by the total number of reads covering that cytosine, which was also equal to the mC/C ratio at each reference cytosine. We also used CGmapTools software to determine the average C-base methylation levels in various types and recalculate the distribution ratio of mC in different samples^51^. With MethGo software, we determined the copy number change for each sample gene^50^. We used the circlize package to plot the distribution of mC sites, differentially methylated regions (DMRs), and copy number variations (CNVs) on the genome^52^.

### Whole-genome bisulfite sequencing

Five gram *C. enshiensis* leaf samples were used for DNA extraction. The qualified DNA was fragmented with an ultrasonic disruptor (Bioruptor) to an average size of ~300–500 bp. The EZ DNA Methylation-Gold™ Kit (Zymo Research Corp., Cat# D5005) was used for bisulfite conversion of DNA and amplification by PCR, and the Pico Methyl-Seq™ Library Prep Kit (Cat# D5455, D5456) was used for post-bisulfite library preparation and genome-wide bisulfite sequencing. The high-quality library had a DNA fragment distribution of ~300 bp. Sequencing on the Illumina 150PE sequencing platform yielded a 30× sequencing depth.

### Genome assembly using PacBio long reads

To assemble contig sequences using long-read data, the software Falcon v0.30^53^ was used with the default parameters. The genome assembly was performed with the following steps in Falcon: first, daligner was used to generate read alignments, and consensus reads were generated. Then, the overlaps among the error-corrected reads were identified by daligner. Finally, a directed string graph was constructed from the overlap data, and the contig paths were resolved by the string graph. The assembled genome sequence was first polished with arrow using PacBio long reads and then by Pilon^54^ with Illumina sequencing data.

### In situ Hi-C library construction and chromosome assembly using Hi-C data

We used Hi-C analysis, which has been demonstrated to be effective in scaffolding, to organize the contigs into chromosomes^55^. The same *C. enshiensis* individual was used for library construction and Hi-C analysis, as described previously^56^. The library was sequenced with the 150PE mode on the Illumina HiSeq X Ten platform (San Diego, CA, USA), yielding 46.54 Gb paired-end reads, and 45.43 Gb filtered reads were used for the following Hi-C analysis. The paired reads were mapped separately to the *C. enshiensis* genome assembly with Bowtie^57^. To increase the interactive Hi-C read ratio, an iterative mapping strategy was used, as reported previously. Only read pairs with both ends uniquely mapped were used for further analysis. Self-ligation, nonligation, and other types of invalid reads, including starting near the rsite, PCR amplification, random breaks, large/small fragments, and extreme fragments, were filtered out of the read pair alignment results by Hi-C as described previously^56^. Through tracking restriction sites, contact counts among the contigs were calculated and normalized. By clustering the contigs using a contig contact frequency matrix, we were also able to correct some minor errors in the Falcon assembly results. Contigs with errors were corrected by breaking them into shorter contigs.

Uniquely mapped read pairs were used for clustering, ordering, and orienting the contigs to construct chromosomes using Lachesis, which employs an agglomerative hierarchical clustering method^57^.

### Genome annotation

Repeat sequences were annotated by Repbase^58^ and a de novo repeat library. The Repbase library was downloaded from http://www.girinst.org/repbase, and the de novo repeat library was constructed by using RepeatModeler (version open−1.0.8, http://repeatmasker.org/RepeatModeler), Piler^59^, RepeatScout^60^, Tandem Repeat Finder (TRF)^61^, and LTRFINDER^62^. RepeatMasker (http://www.repeatmasker.org) was used to identify repetitive elements in the *C. enshiensis* de novo repeat library and the Repbase library.

The protein-coding gene annotation incorporated homology prediction, ab initio prediction, and full-length transcriptome prediction based on third-generation sequencing. For the homology-based method, the protein sequences of *A. thaliana*, *B. napus*, *Brassica oleracea*, *C. hirsuta*, and *Brassica rapa* were downloaded from Ensembl (http://plants.ensembl.org/index.html) and mapped to the *C. enshiensis* genome using TBLASTN^63^. Augustus^64^ and GlimmerHMM^65^ were used for ab initio prediction of genes in the repeat-masked genome. Full-length transcripts obtained using Iso-Seq were mapped to the genome using GMAP^66^, and then, the TransDecoder program^67^ was used to predict open reading frames (ORFs) in the transcripts to define putative coding sequences (CDSs). Finally, we predicted a total of 52,725 protein-coding genes in the *C. enshiensis* genome by integrating all the gene models with MAKER^8^, following extensive and careful manual inspections. The gene number, gene length distribution, CDS length distribution, exon length distribution, and intron length distribution were comparable to those in other species (Supplementary Fig. S18). Gene functional annotations were assigned through sequence homology searches. Protein databases (Swiss-Prot, TrEMBL, KEGG, InterPro, and GO) were used to perform functional annotation of proteins with BLAST2GO^68^. BUSCO^7^ was used to determine the sensitivity of our predicted protein-coding genes.

ncRNAs were annotated using various software packages and databases. tRNAscan-SE^69^ software was used to find the tRNA sequences with the eukaryote parameters. We searched the Rfam^70^ database using Infernal cmscan^71^ to detect microRNAs, rRNAs, small nuclear RNAs, and small nucleolar RNAs.

### Analysis of gene family evolution

Protein sets were collected from 9 sequenced plant species: *A. thaliana*, *Arabidopsis lyrata*, *B. rapa*, *B. oleracea*, *Raphanus sativus*, *Eutrema salsugineum*, *C. hirsuta*, *Capsella rubella*, and *Carica papaya*. All-against-all BLASTP^72^ was used to identify homologous genes from the different species.

We clustered the protein sequences of each species based on sequence similarity with OrthoMCL^73^ to identify orthologous genes (Supplementary Figs. S19 and S20). An evolutionary tree was constructed based on the shared single-copy orthologous genes obtained from gene family clustering. Muscle^74^ was used to perform multiple sequence alignments of the genes in each single-copy homologous gene family. Then, the results of the multiple sequence alignments were combined and converted into a supergene alignment in phylip format. A total of 1124 single-copy orthologous genes were retained after length filtering, and RAxML^75^ was used to build a phylogenetic tree by maximum likelihood.

Using the constructed evolutionary tree combined with the TimeTree database (www.timetrees.org) and the literature, we obtained time correction points. A Bayesian molecular clock and penalized likelihood were used to estimate divergence times by MCMCTREE in PAML^76^.

CAFÉ^77^ was used to simulate the gene family expansion and contraction events of each lineage on the evolutionary tree. The PAML software package was used to detect whether each gene was under positive selection with a branch-site model according to each shared single-copy orthologous gene family. Statistical significance was tested with Fisher’s exact test corrected for multiple testing by the Bonferroni method and a false discovery rate (FDR) threshold of 0.05 (Supplementary Fig. S21).

### WGD in *C. enshiensis*

We used *K*s (synonymous substitution rate) estimation to detect WGD events in the *C. enshiensis* genome. First, the all-versus-all BLASTP^72^ method (*E*-value < 1e^–5^) was used to detect orthologous genes in the different species. Then, syntenic paralogous blocks were identified with MCSCAN^78^ (Supplementary Fig. S22). We extracted all the paralogous and orthologous gene pairs from the syntenic blocks in those species to further calculate their *K*s values with the PAML yn00 NG model^76^, and the divergence time was calculated by the formula *K*s/2*r*. Finally, the potential WGD events in each genome were evaluated based on their *K*s distribution (Supplementary Fig. S2).

### In situ Hi-C library construction

Plant leaf samples weighing ~2 g were fixed with 2% formaldehyde, extracted, lysed with 0.1% SDS, digested with 200 U MboI (NEB) and labeled with biotinylated cytosine nucleotides by biotin-14-dCTP (TriLINK). Blunt end ligation was carried out with T4 DNA ligase, and the crosslinking was reversed with 200 µg/mL proteinase K (Thermo Fisher). The purified DNA was sheared to a length of ~400 bp. Point ligation junctions were pulled down with Dynabeads^®^ MyOne™ Streptavidin C1 (Thermo Fisher) according to the manufacturer’s instructions. The Hi-C library for Illumina sequencing was prepared using the NEBNext^®^ Ultra™ II DNA Library Prep Kit for Illumina (NEB) according to the manufacturer’s instructions. Fragments between 400 and 600 bp were subjected to paired-end sequencing on the Illumina HiSeq X Ten platform (San Diego, CA, USA) in the 150PE mode. Two replicates were generated for each group of samples.

### Hi-C analysis

#### Chromosome assembly using Hi-C data

First, 46.54 Gb raw read pairs were generated from the Hi-C library and mapped to the polished genome using BWA (bwa-0.7.17)^79^ with the default parameters. Paired reads with mates mapped to a different contig (or scaffold) were used to perform Hi-C-associated scaffolding. We then successfully clustered 2267 contigs (443.45 Mbp in length) into 16 groups by the agglomerative hierarchical clustering method in Lachesis^80^. Lachesis was further applied to order and orient the clustered contigs. Then Juicebox (v1.8.8)^81^ was used to correct assembly errors by eye. Overall, 1089 contigs were successfully assembled to yield a total length of 383.27 Mbp. Finally, we obtained the first chromosome-level high-quality assembly of the *C. enshiensis* genome, in which the chromosomal lengths ranged from 15.82 Mb to 29.81 Mb, and the assembly encompassed 86.65% of the total sequence.

#### Construction of the contact map

After quality filtering using Trimmomatic (version 0.38)^82^, the clean Hi-C data of two biological replicates for leaves and two biological replicates for roots were iteratively mapped to the genome using the ICE software package (version 1f8815d0cc9e). Dangling ends and other unusable data were filtered, and the remaining valid pairs were used to analyze the correlation coefficients between the two biological replicates for each sample using QuASAR-Rep analysis^83^ (3DChromatin-ReplicateQC v0.0.1). Then, we pooled the data from each pair of replicates for further analysis.

A Hi-C map is a list of DNA–DNA contacts produced by a Hi-C experiment. The valid pairs after pooling were binned into 500-kb (200, 100, 40, 20, 10, and 5 kb) nonoverlapping genomic intervals to generate contact maps. Raw Hi-C contact maps can contain many biases, such as mappability, GC content, and uneven distribution of restriction enzyme sites. Here, the contact maps were normalized using an iterative normalization method to eliminate systematic biases.

#### Map resolution analysis

We defined the “matrix resolution” of the Hi-C map as the locus size used to construct a particular contact matrix and the “map resolution” as the smallest locus size such that 80% of the loci had at least 1000 contacts^29^. The map resolution was meant to reflect the finest scale, at which local features could reliably be discerned.

#### Compartment analysis

Chromatin compartments are defined as groups of domains located on the same chromosome or different chromosomes that display increased interactions with each other. In heatmaps generated from 200 kb bins, this is visible as a specific plaid pattern, in which the alternating blocks of high and low interaction frequencies represent the A and B compartments. PCA readily identifies these compartments, which tend to be captured by the first component. For each arm on an individual chromosome, genomic bins with a positive or negative first eigenvector (PC1) were assigned to the A or B compartment. The active A compartment contained gene-dense euchromatic regions, whereas the inactive B compartment contained gene-poor heterochromatic regions.

#### TAD analysis

TADs are contiguous regions that display high levels of self-association and are separated from adjacent regions by distinct boundaries. The locations of TADs can be determined when interaction data are binned at 40 kb. We used an insulation score algorithm^22^ to identify the locations of TAD boundaries in each sample and to determine the locations and number of TADs.

#### Calculation of intra- and interchromosomal interactions

The contacts between the 10-kb bins of intrachromosomal and interchromosomal interactions of each sample were examined by Ay’s Fit-Hi-C software (v1.0.1)^84^ (with parameter settings of L 20,000 –U 2,000,000–p 2 –b200) to calculate the corresponding cumulative probability (*P* value) and FDR (*q* value). After this calculation, the interactions in which both the *P* value and *q* value were < 0.01, and the contact count was > 2 were identified as significant interactions.

## Supplementary information

Supplementary Tables S1-S26

Supplementary Figs. S1-S22

## Data Availability

The sequence reads are available at the NCBI Sequence Read Archive (SRA) as Bioproject PRJNA565347. The genome assembly sequences and gene annotations have been deposited into the Genome Warehouse BIG Data Center under accession number PRJCA002827.
