# Author Correction: The *Cardamine enshiensis* genome reveals whole genome duplication and insight into selenium hyperaccumulation and tolerance

**DOI:** 10.1038/s41421-021-00330-w

**Published:** 2021-09-23

**Authors:** Chuying Huang, Hongqing Yin, Xibiao Yang, Yuan Gao, Tuo Li, Bo Wu, Meng Ren, Zixiong Zhang, Jun Ding, Jianhua Gao, Dan Wen, Xingzhi Ye, Ling Liu, Huan Wang, Guogen Sun, Yi Zou, Nansheng Chen, Li Wang

**Affiliations:** 1 Hubei Minzu University Affiliated Enshi Clinical Medical School, The Central Hospital of Enshi Tujia and Miao Autonomous Prefecture, Enshi, Hubei China; 2 Hubei Selenium and Human Health Institute, Enshi, Hubei China; 3 Hubei Selenium Industrial Technology Research Institute, Enshi Autonomous Prefecture Academy of Agriculture Sciences, Enshi, Hubei China; 4 Department of Radiology, West China Hospital of Sichuan University, Chengdu, Sichuan China; 5 Department of Chemistry and Molecular Biology, University of Gothenburg, SE 405 30 Gothenburg, Sweden; 6 Center for Bioinformatics and Computational Biology, and the Institute of Biomedical Sciences, School of Life Sciences, East China Normal University, Shanghai, China; 7 South China Potato Research Center, Enshi Autonomous Prefecture Academy of Agricultural Sciences, Enshi, Hubei China; 8 Bureau of Agricultural & Rural Affairs of Enshi Tujia and Miao Autonomous Prefecture, Enshi, Hubei China; 9 Wuhan Frasergen Bioinformatics Co., Ltd., Wuhan, Hubei China; 10 Institute of Oceanology, Chinese Academy of Sciences, Qingdao, Shandong China; 11 Department of Molecular Biology and Biochemistry, Simon Fraser University, Burnaby, Canada

**Keywords:** Plant molecular biology, Plant sciences

Correction to: *Cell Discovery* (2021) 7:62

10.1038/s41421-021-00286-x Published online 10 August 2021

In the original publication of this article^1^ one of the author’s name was incorrect. In this correction article, the correct and incorrect name are indicated.

Previous incorrect name:

Hongqin Ying

Corrected name:

Hongqing Yin

Previous incorrect name:

These authors contributed equally: Chuying Huang, Hongqin Ying, Xibiao Yang, Li Wang.

Corrected name:

These authors contributed equally: Chuying Huang, Hongqing Yin, Xibiao Yang, Li Wang.
