# Supplementary material for: The Cardamine enshiensis genome reveals whole genome duplication and insight into selenium hyperaccumulation and tolerance
Source: Cell Discov. 2021 Aug 10;7:62. doi: 10.1038/s41421-021-00286-x (PMC8352907; doi:10.1038/s41421-021-00286-x)
Supplement: Supplementary file 2 — Supplementary Figs. S1-S22 [file 41421_2021_286_MOESM2_ESM.pdf]

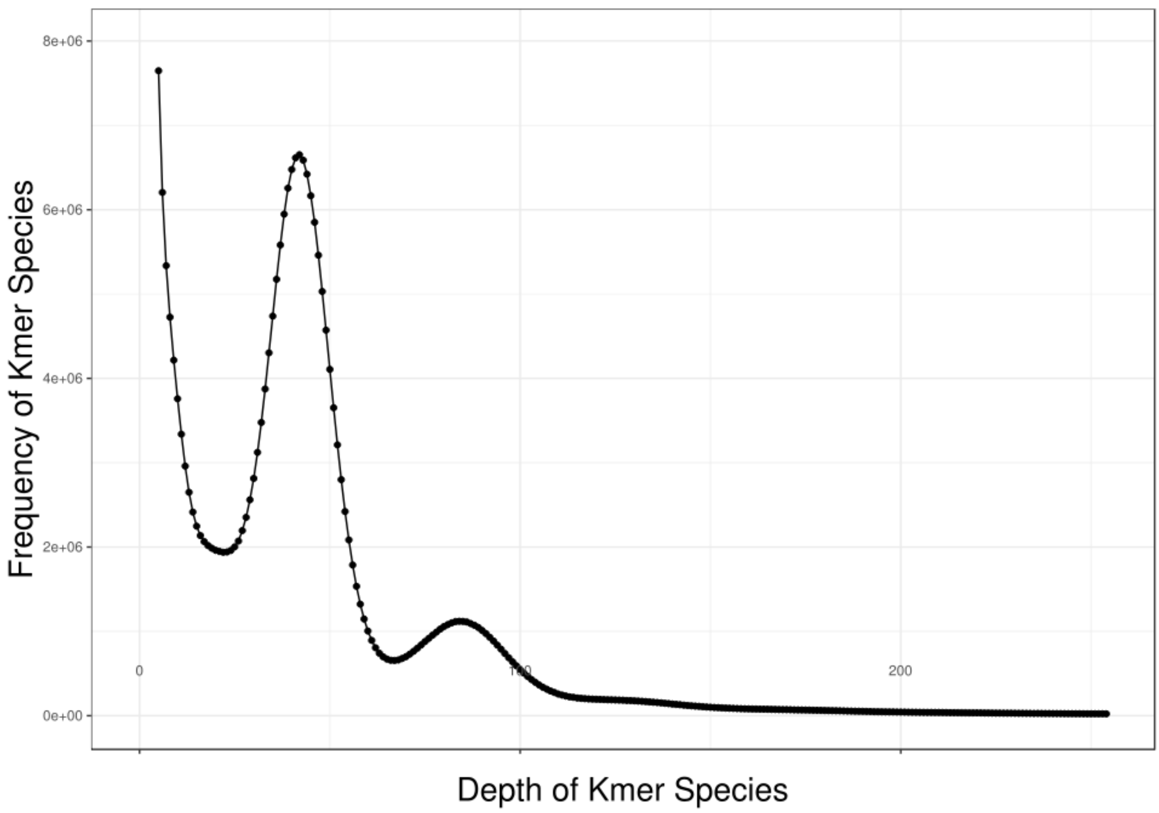

Supplementary Fig. S1 Kmer frequency distributions from base error-corrected reads. Using a Kmer size of 17, there was a frequency peak value at 42, which was used for genome size estimation.

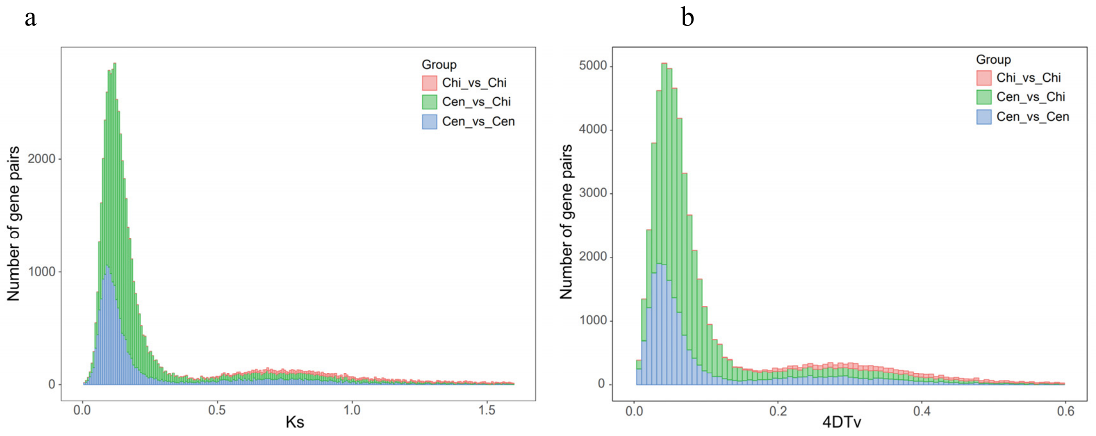

Supplementary Fig. S2 a Histogram distribution of Ks (synonymous substitution rate) for orthologous gene pairs in *C. enshiensis* and *C. hirsuta*. **b** Distances corresponding to the *C. enshiensis* whole-genome duplication events were delineated based on discrete peaks in 4DTV distributions.

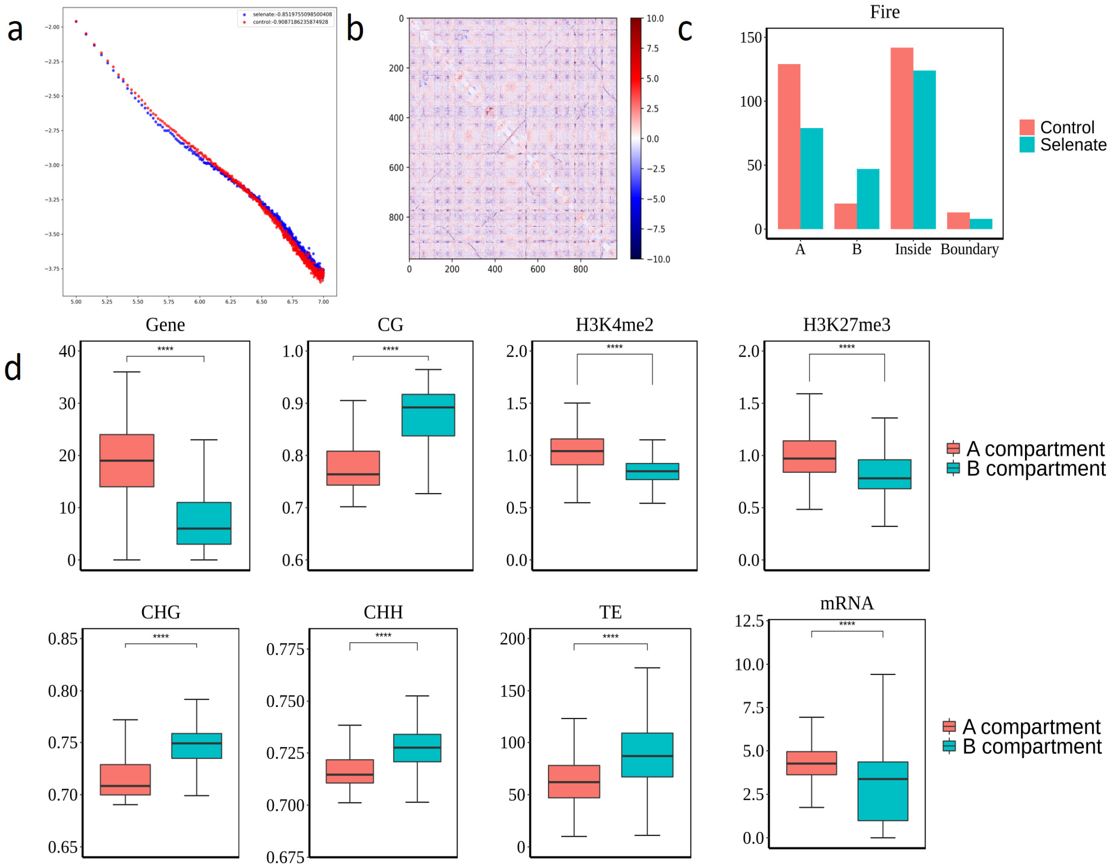

Supplementary Fig. S3 Se affects the 3D genome architecture of *C. enshiensis.* a Whole-genome distance-interaction frequency diagram with a 400-kb resolution between the samples. Color: Interaction attenuation curve of different samples (Red: control, Blue: selenate); horizontal axis: relative distance between different sites on the chromosome; vertical axis: interaction frequency. **b** The interaction subtraction matrix between the two samples across the whole genome at a 400-kb resolution. **c** FIREs were identified at a 10-kb resolution in the respective chromatin structural units, including A and B compartments and TAD boundaries and interior regions. **d** Genomic and epigenetic features of the *C. enshiensis* A/B compartments.

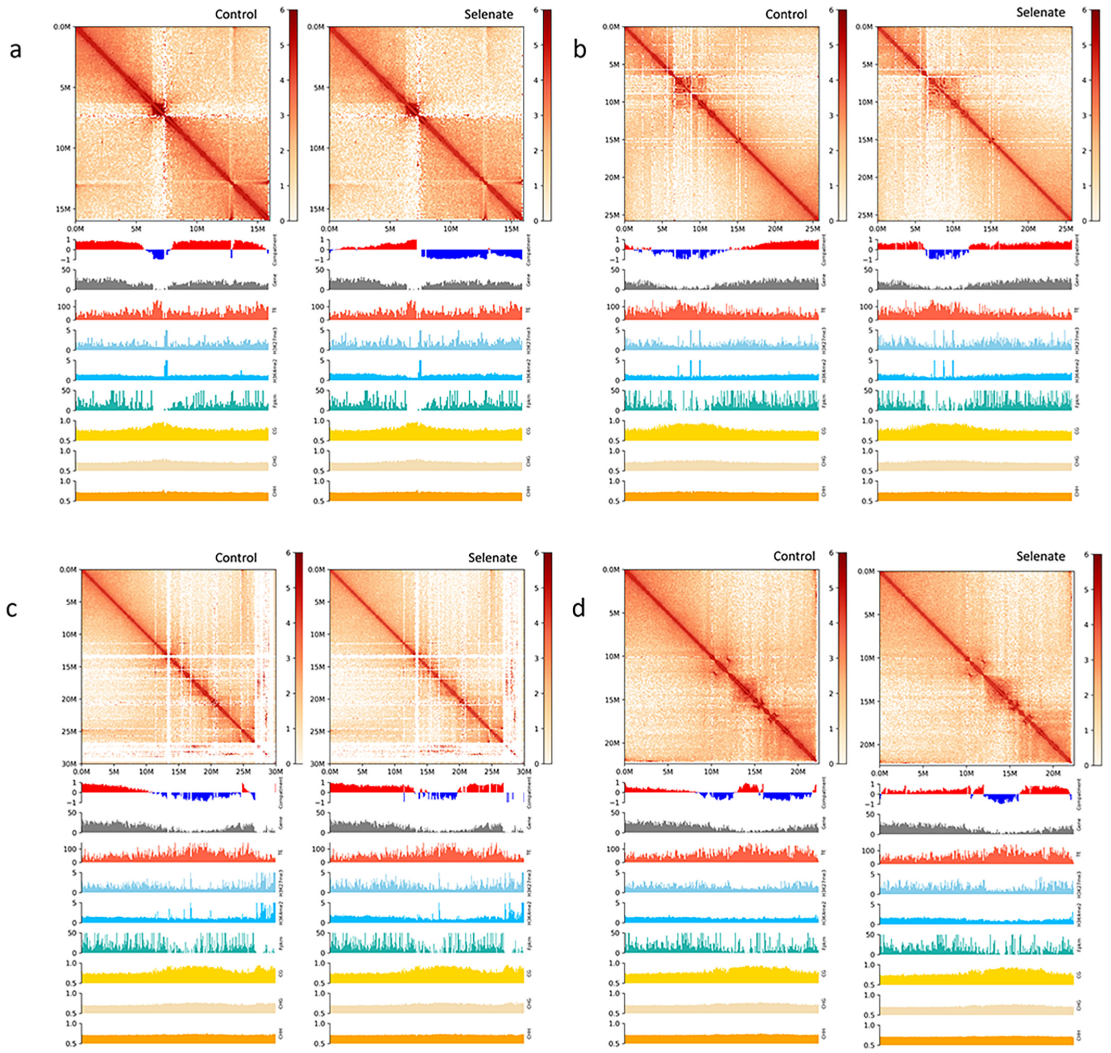

Supplementary Fig. S4 Se remodels the *C. enshiensis* A/B compartments. a-d Hi-C interaction map and colocalization of the genomic composition and various epigenetic marks on Chr2 (**a**), Chr8 (**b**), Chr9 (**c**), Chr10 (**d**) (control and selenate group). The heatmap denotes the intrachromosomal Hi-C interaction frequencies among the pairwise 100-kb bins shown on the top. The PCA eigenvectors of the A and B compartments and genomic and epigenetic feature tracks are shown below in separate 100-kb bins, including abundances of genes and TEs, different histone modifications, mRNA expression levels (normalized by FPKM), and DNA methylation (in CG, CHG and CHH contexts).

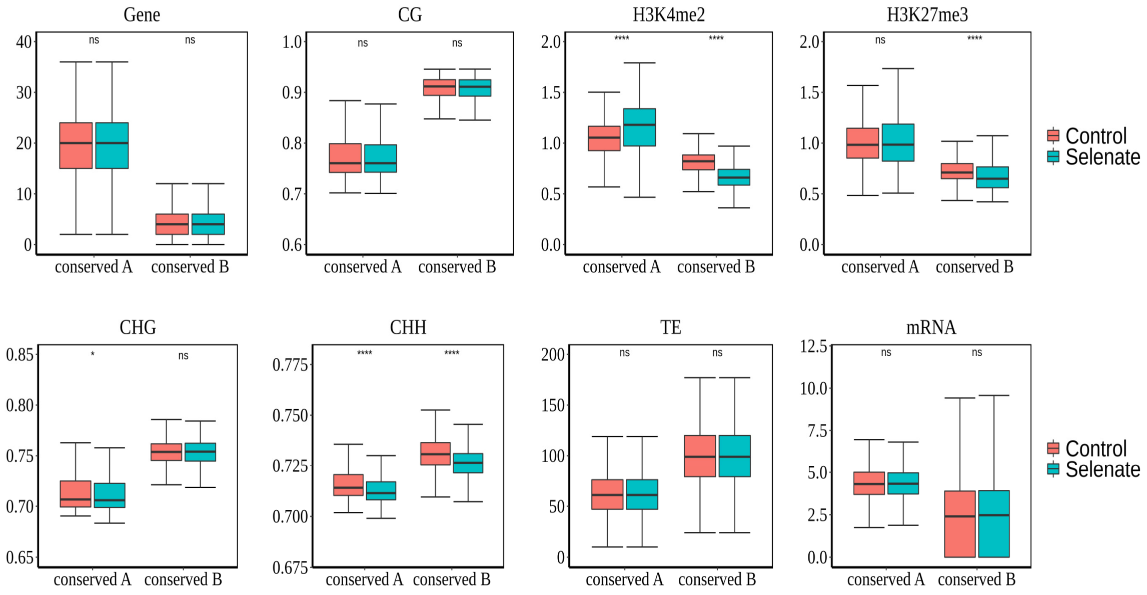

Supplementary Fig. S5 The effect of selenate on the A/B compartments. Conserved A compartment domains had a higher gene density and activating epigenetic markers, while conserved B compartment domains and differential compartment domains were associated with increased TE densities and decreased repressive epigenetic markers.

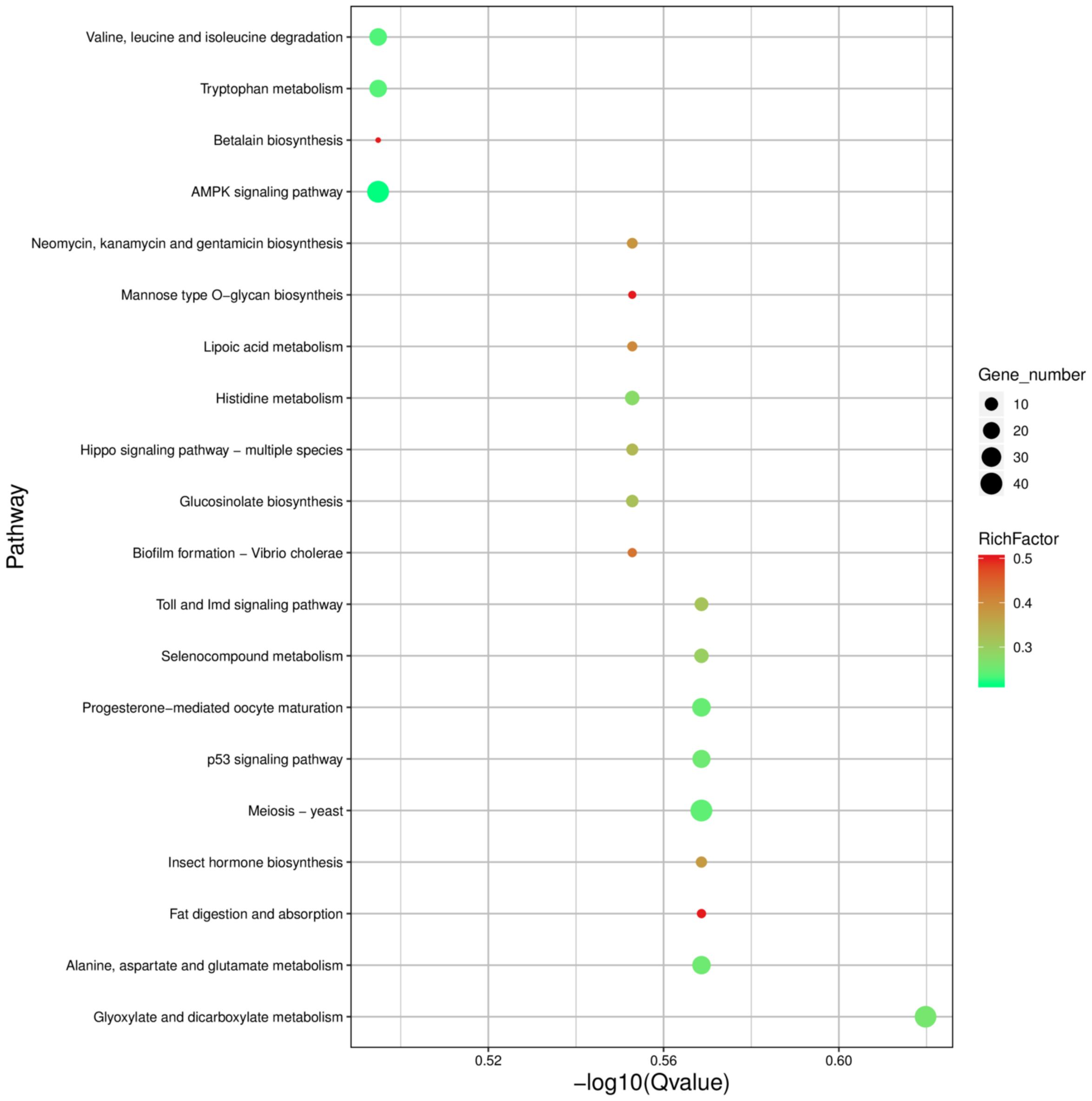

Supplementary Fig. S6 Bubble pattern of enriched pathways in the differential and conserved compartment domains. The Y-axis represents the name of the pathway or function, and the X-axis represents the q value. The size of the bubble represents the number of genes in a signaling pathway or involved in a function. The color of the bubble indicates the ratio of the number of genes enriched in the pathway to the total number of annotated genes.

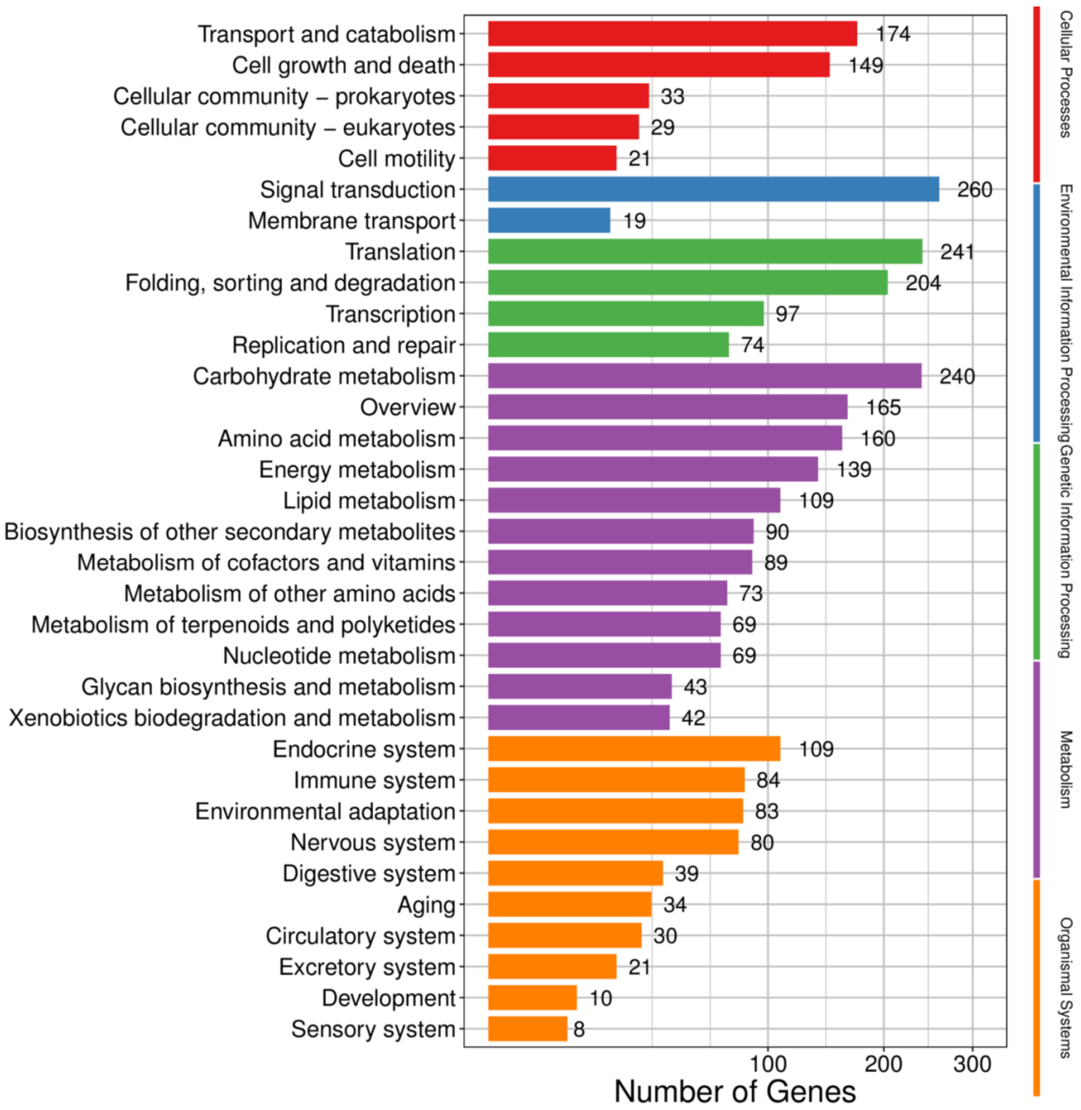

Supplementary Fig. S7 Functional classification of KEGG pathways in the differential and conserved compartment domains. The KEGG pathways were classified into five main categories: A, Cellular Processes; B, Environmental Information Processing; C, Genetic Information Processing; D, Metabolism; E, Organismal Systems. The y-axis indicates the name of the KEGG metabolic pathways. The x-axis indicates the percentage of genes annotated to that pathway compared to the total number of annotated genes.

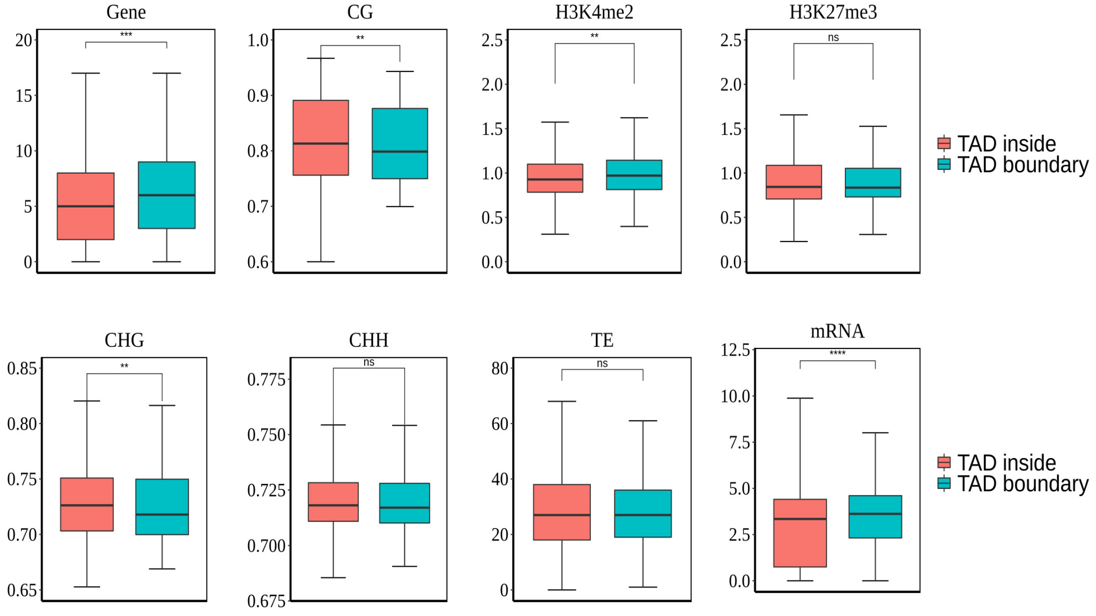

Supplementary Fig. S8 Genomic and epigenetic features of the *C. enshiensis* TAD boundaries. TAD boundaries had lower gene densities and higher gene expression levels compared to TAD interior regions.

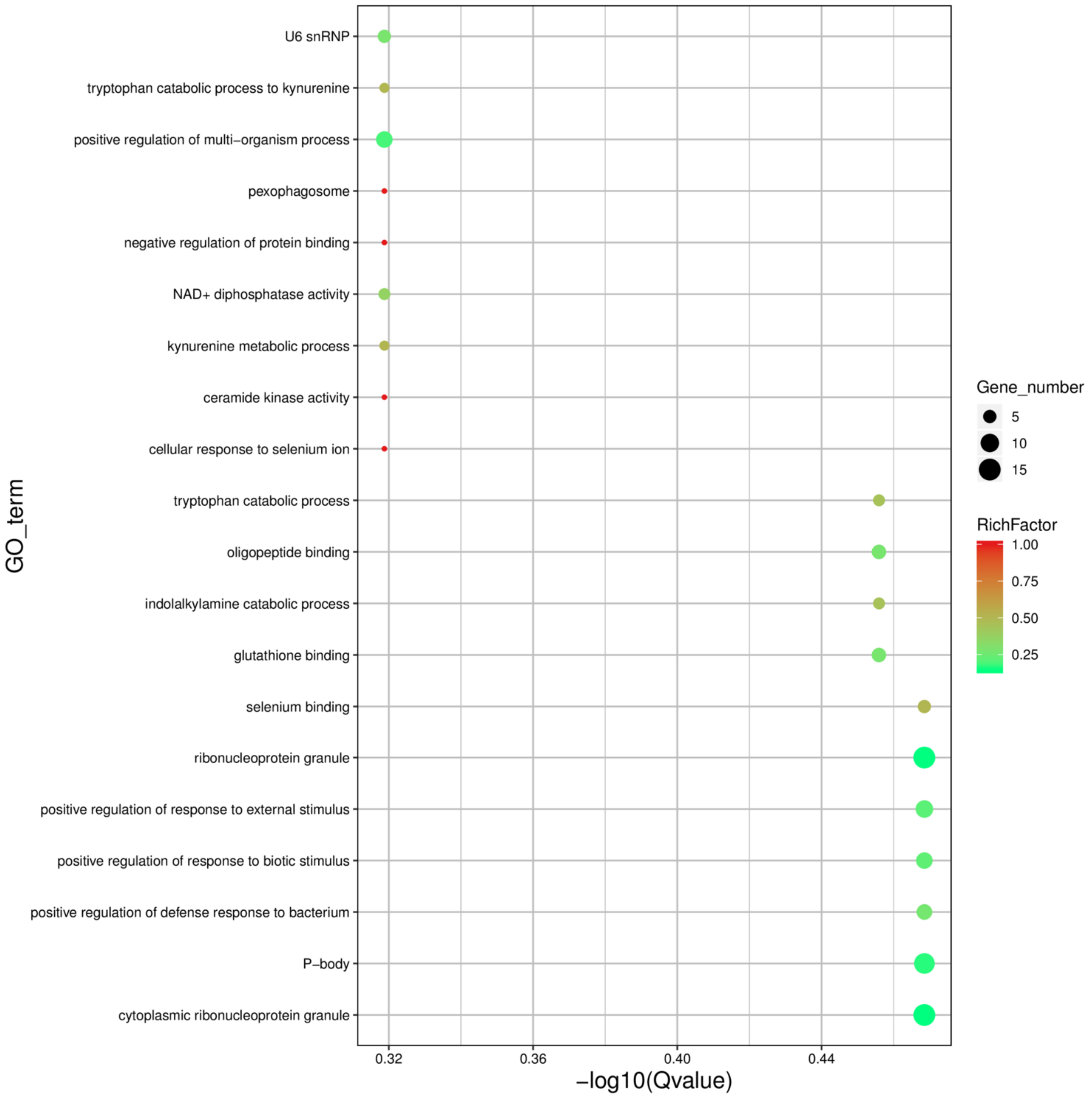

Supplementary Fig. S9 Bubble pattern of enriched pathway in the Insulation region between samples. The Y-axis represents the name of GO term, and the X-axis represents the q value. The size of the bubble represents the number of genes in a signaling pathway or involved in a function. The color of the bubble indicates the ratio of the number of genes enriched in the pathway to the total number of annotated genes.

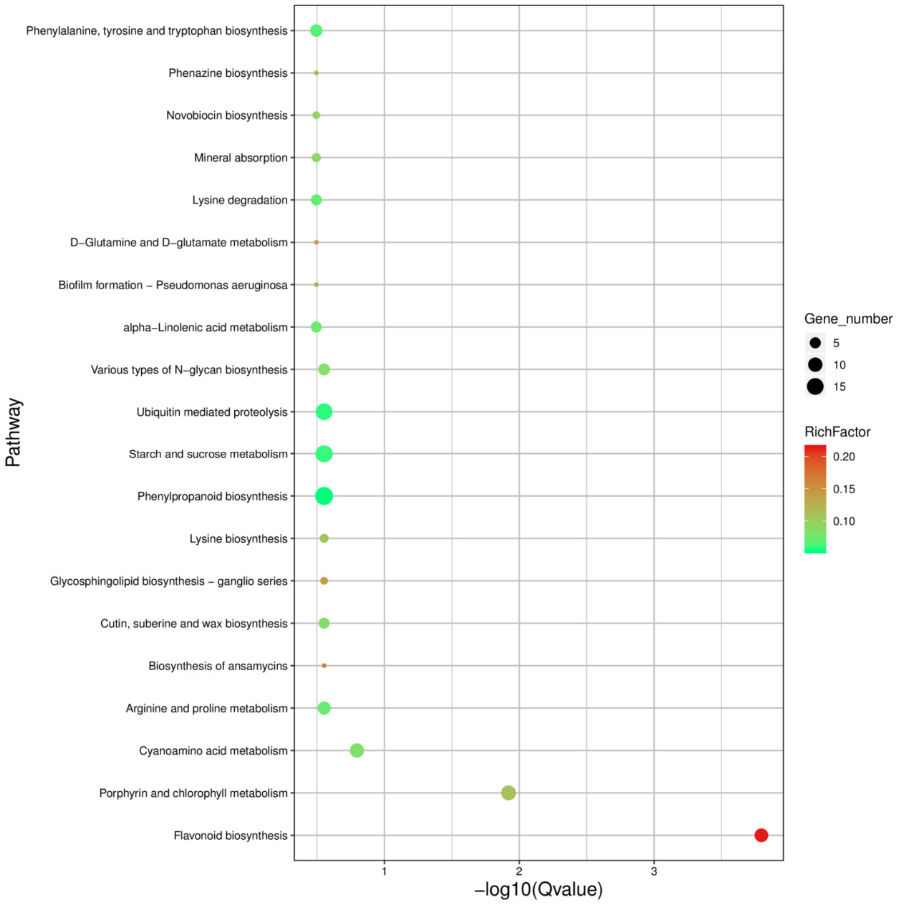

Supplementary Fig. S10 KEGG pathway analysis of differentially expressed genes in the Insulation region between samples. The advanced bubble chart shows the enrichment of the differentially expressed genes in signaling pathways. The Y-axis labels represent pathways, and the X-axis represents the rich factor. The size and color of each bubble represent the number of differentially expressed genes enriched in the pathway and the enrichment significance, respectively.

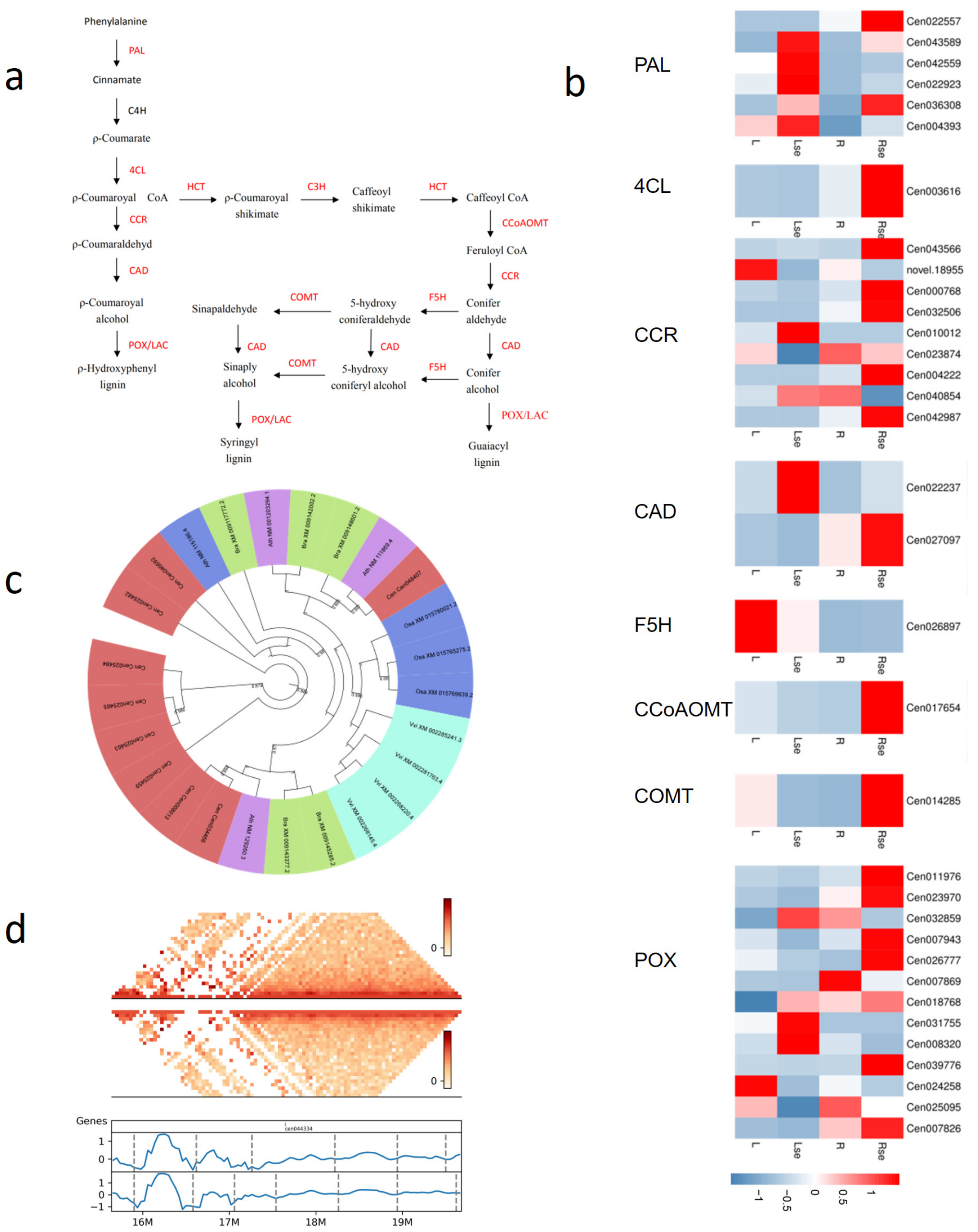

Supplementary Fig. S11 Lignin plays an important role in Se tolerance and accumulation. a The reported pathway and genes that regulate the biosynthesis of lignin. **b** Expression analysis of lignin biosynthetic genes. **c** Phylogenetic tree of the PAL gene family, which produces a key enzyme in the lignin and flavonoid biosynthetic pathways. **d** The Hi-C interaction matrix (cen044334, chr12, 17603950-17609563, 2 Mb range upstream and downstream of the PAL gene) shows interactions and TAD signals (40 kb resolution). Top: Hi-C interaction matrix, middle: TAD boundaries (vertical bars) and insulation scores. The vertical axis and the blue line in the figure represent the insulation score, and the gray line in the figure shows the TAD boundary.

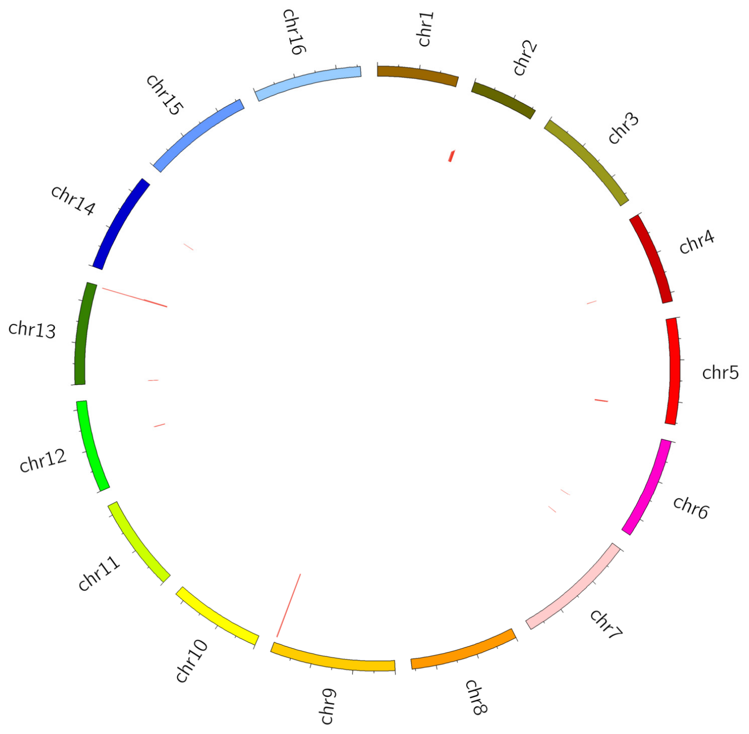

Supplementary Fig. S12 Frequently interacting regions were identified at a 10-kb resolution, and these regions were remarkably enriched on chromosomes 2, 9, and 13.

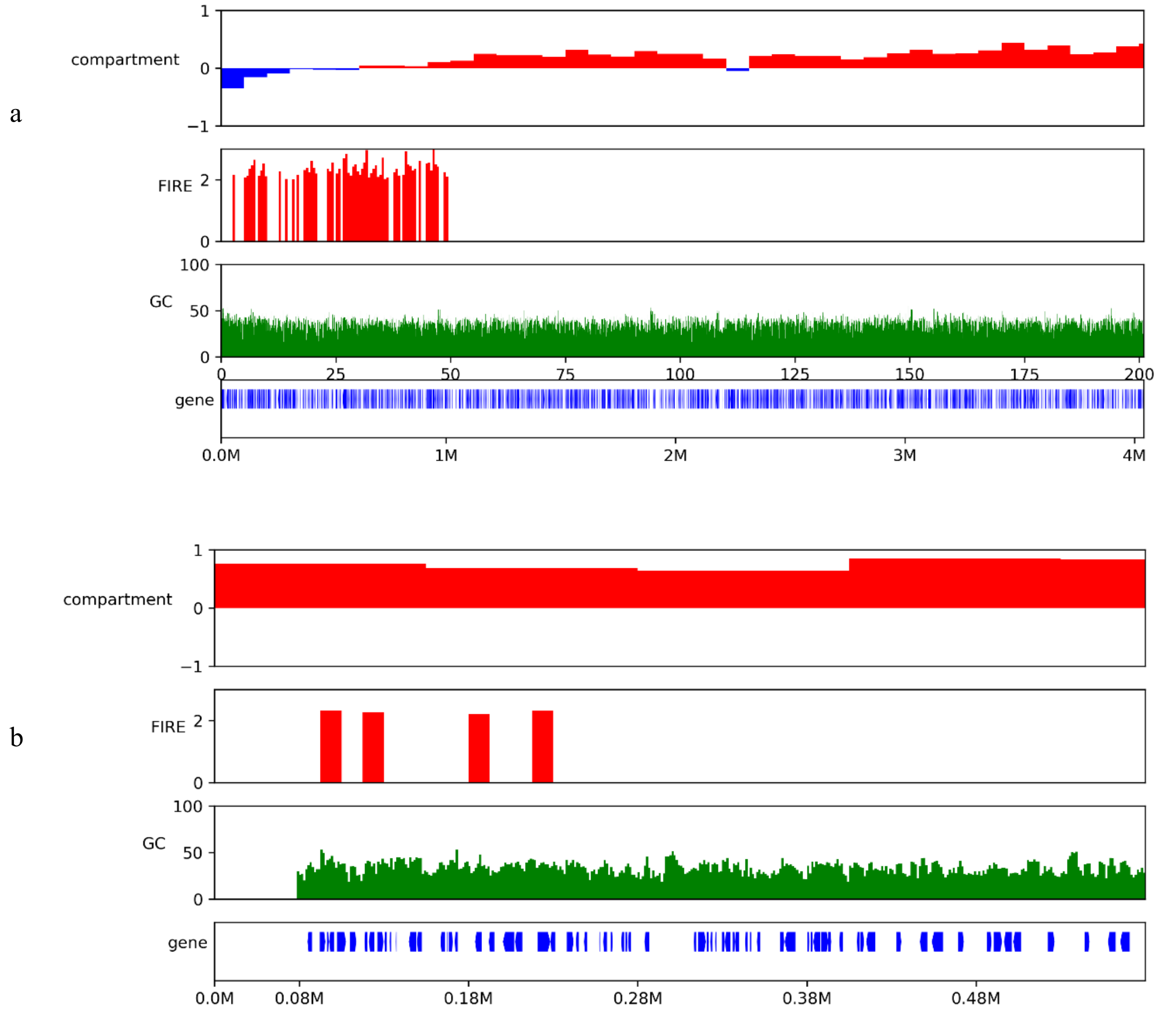

Supplementary Fig. S13 a The compartment, GC and gene distribution in dense areas of FIRE sites on chr2. **b** The compartment, GC and gene distribution in dense areas of FIRE sites on chr13.

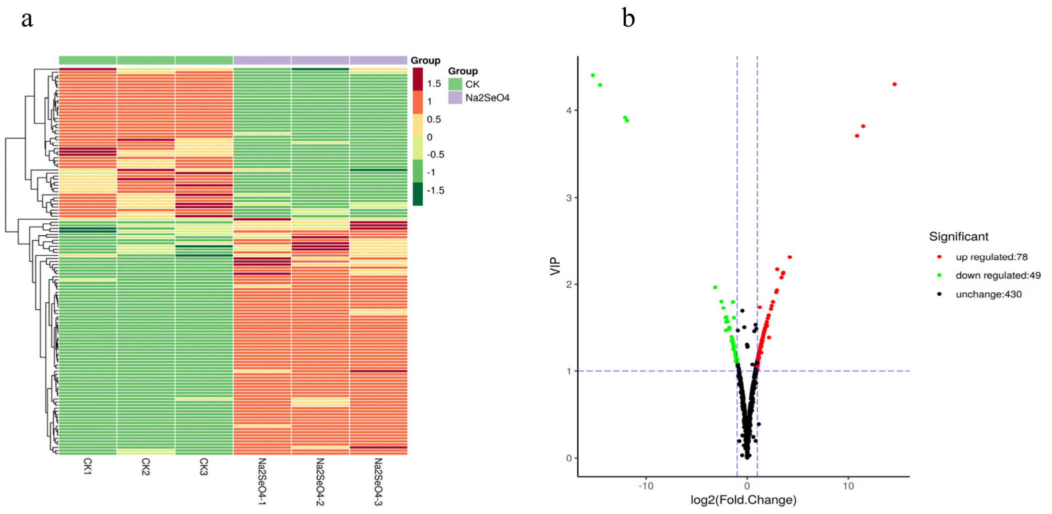

Supplementary Fig. S14 a Cluster heat map. The columns of the microarray heat map represent the samples, and the rows represent the metabolites. Each cell is colorized based on the level of expression of the corresponding metabolite in that sample. **b** Volcano plot showing differences in the expression levels of the metabolites in the two groups. The green dots represent downregulated metabolites, the red dots represent upregulated metabolites, and the black dots represent metabolites without significant differences.

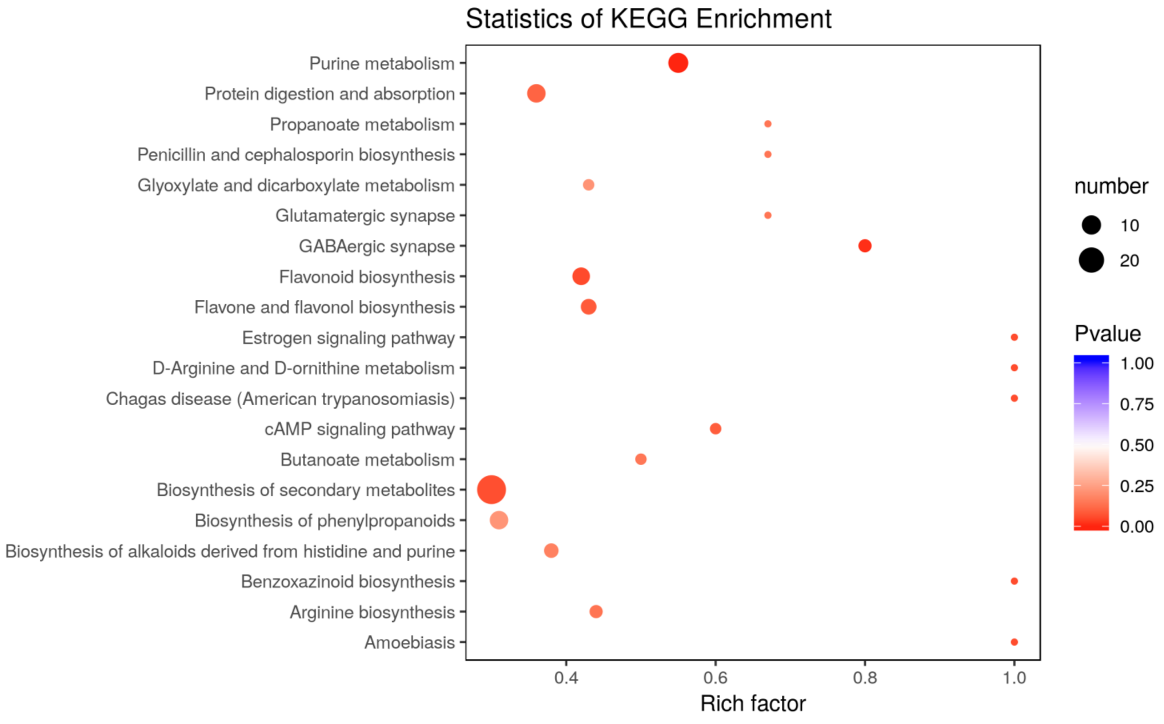

Supplementary Fig. S15 KEGG pathway analysis of differentially expressed metabolites. The advanced bubble chart shows the enrichment of the differentially expressed metabolites in signaling pathways. The Y-axis labels represent pathways, and the X-axis represents the rich factor (the number of differentially expressed genes enriched in the pathway/the number of all genes in the background gene set). The size and color of each bubble represents the number of differentially expressed metabolites enriched in the pathway and the enrichment significance, respectively.

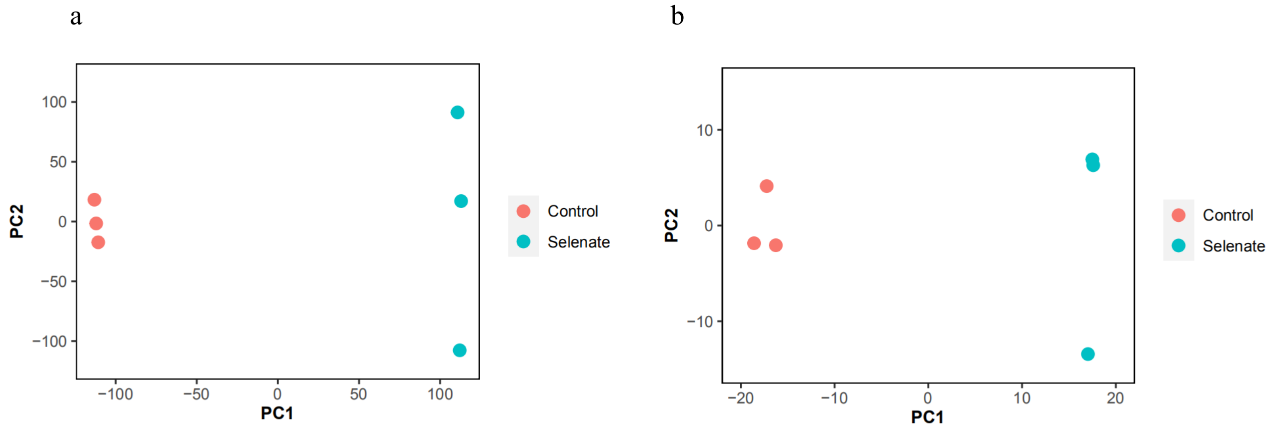

Supplementary Fig. S16 a, b Principle component analyses of 29,671 differentially expressed genes (**a**) and 554 metabolites in the control and 400 μM sodium selenate treatment group (**b**).

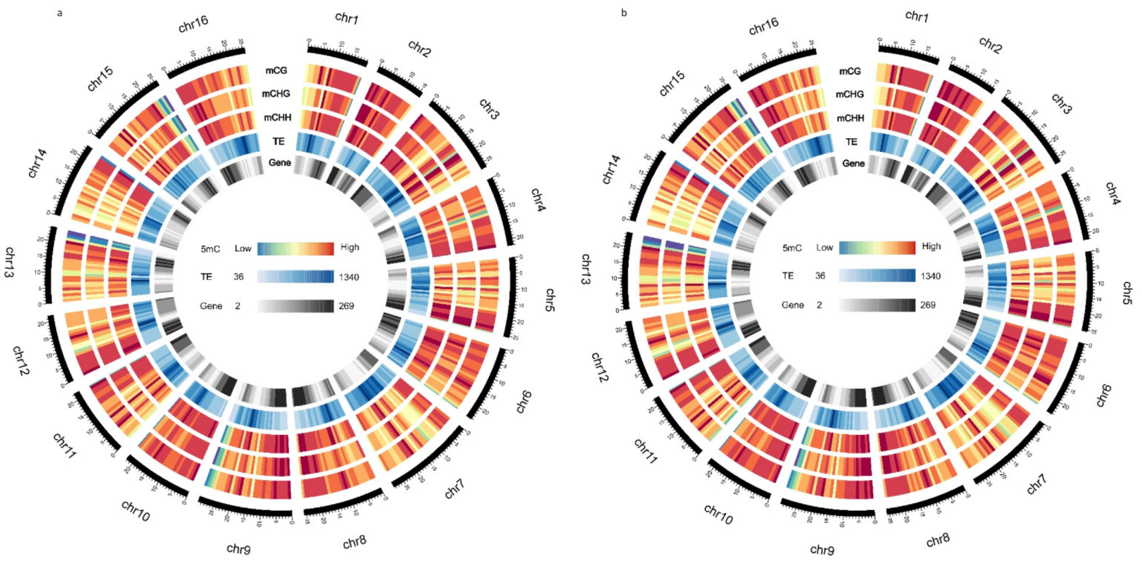

Supplementary Fig. S17 The *C. enshiensis* epigenome. a, b Circos plots of chromosomes in the *C. enshiensis* genome. Track order: density plot of 5mC in the CG, CHG, and CHH contexts; density of transposable elements (TEs) (**a**, control; **b**, selenate).

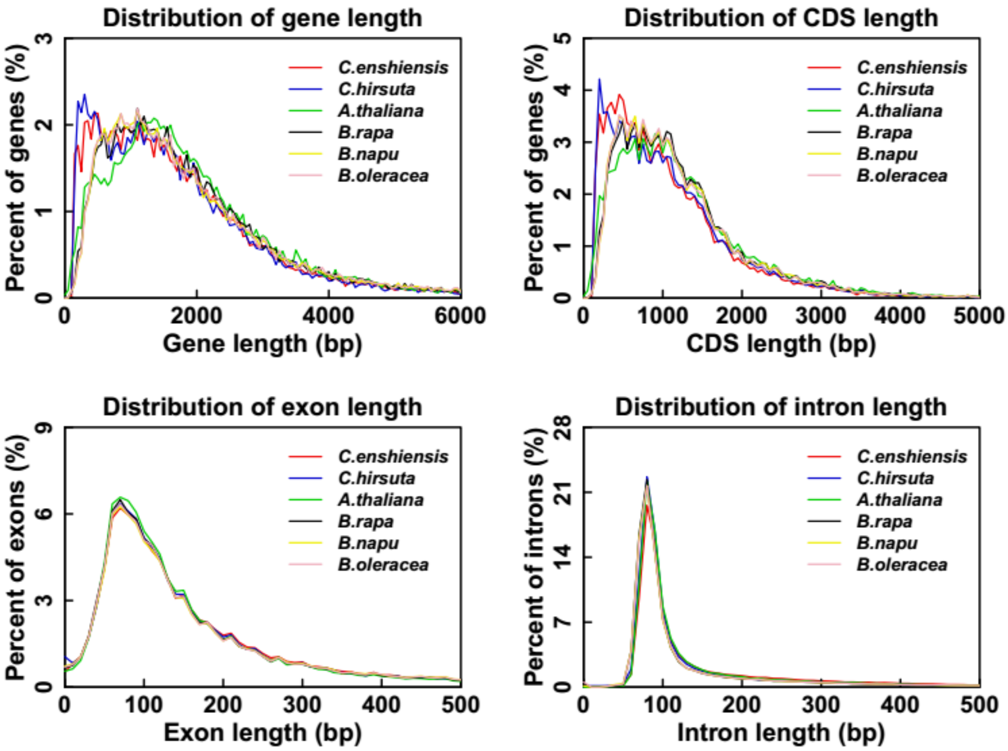

Supplementary Fig. S18 Length distribution comparison of total genes, CDSs, exons and introns of the annotated gene models of *C. enshiensis* and 5 additional species. The length distributions of the total genes, CDSs, exons and introns were compared to those of *C. hirsuta*, *A. thaliana*, *B. rapa*, *B. napus*, and *B. oleracea*. Sharing of gene families between *C. enshiensis* and *A. thaliana*, *A. lyrata*, *B. rapa*, and *B. oleracea*, with *B. oleracea* as an outgroup.

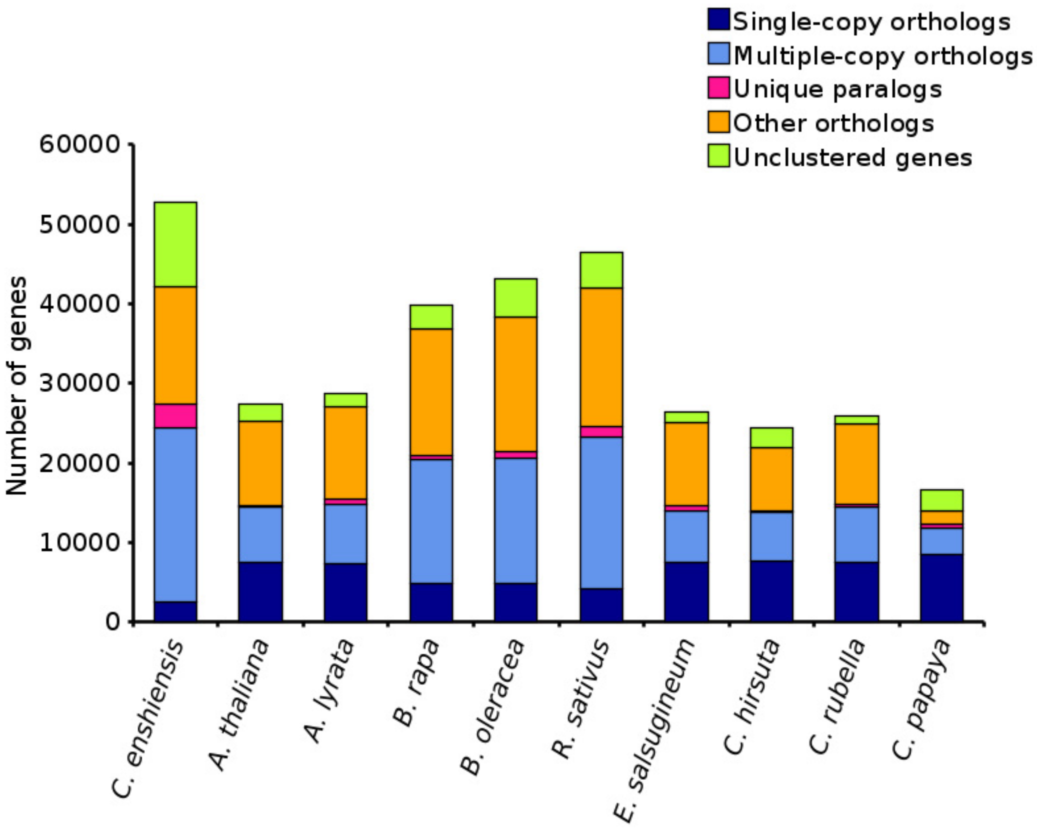

Supplementary Fig. S19 Classification statistics of gene family clustering based on *C. enshiensis* and 9 additional species.

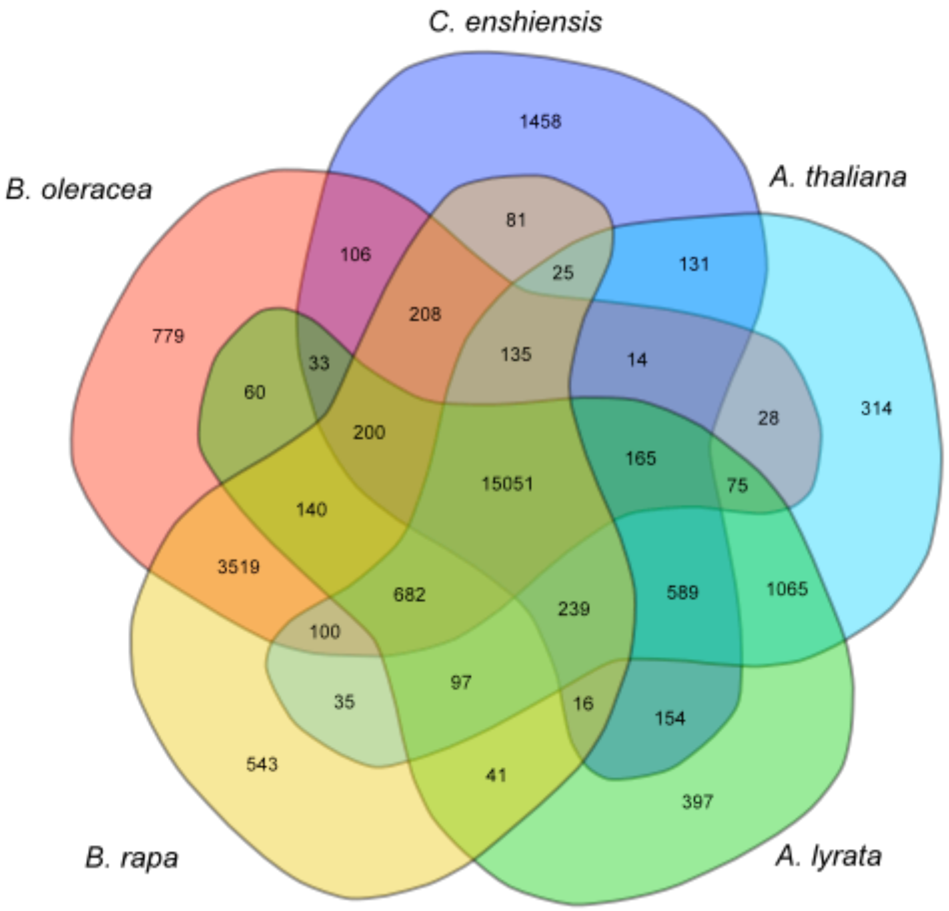

Supplementary Fig. S20 Sharing of gene families by *C.* enshinesis and *A. thaliana*, *A. lyrata*, *B.* rapa, *B.*oleracea, *B.* oleracea as an outgroup.

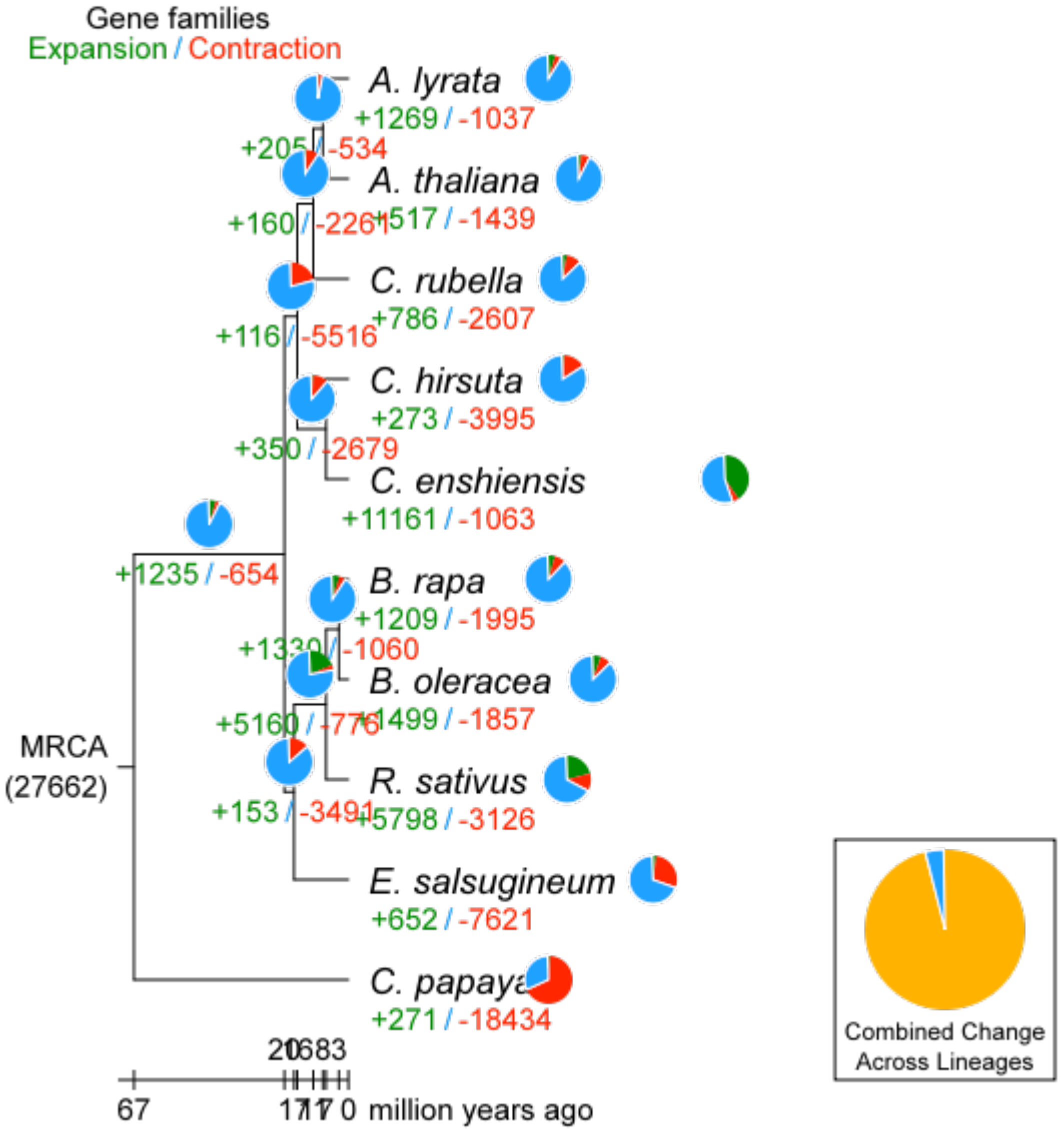

Supplementary Fig. S21 Gene family expansion and contraction analysis of *C. enshiensis* in comparison to 9 other plants. The numbers separated by slashes (expansions/contractions) indicate gene family numbers. There were 27,662 gene families in the most recent common ancestor (MRCA) of all the species. A detailed list of the expanded and contracted gene families and GO/KEGG enrichment results can be found in the supplemental materials (Supplementary Tables 23-26). Through positive selection analysis, a total of 110 positively selected genes were identified (FDR < 0.05).

a b

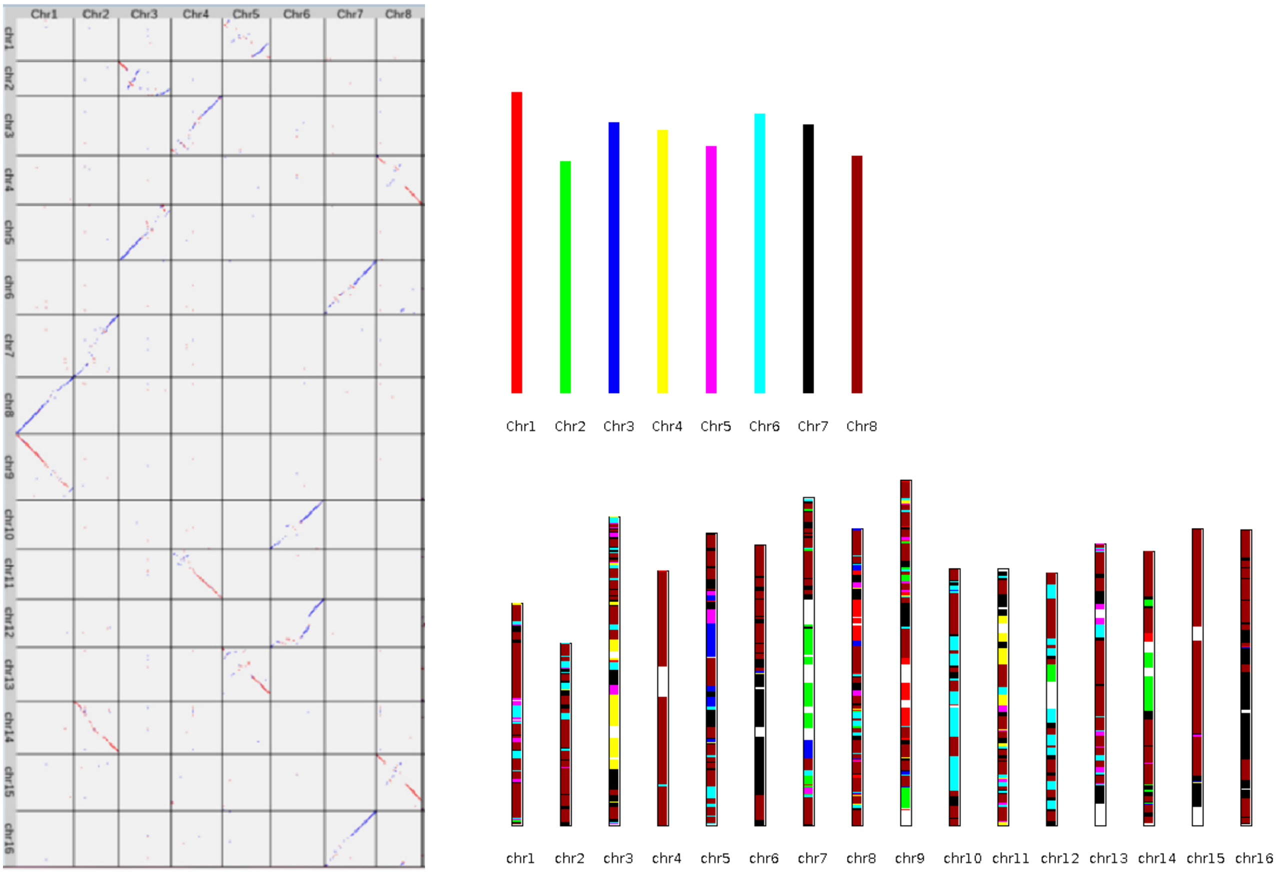

Supplementary Fig. S22 a Dot plot showed a 2:1 syntenic relationship between *C. enshiensis* and *C. hirsuta*. **b** Genome painter image shows gene collinearity between the *C. hirsuta* and *C. enshinesis* genomes. Synteny from paralogs and orthologs was detected by MCScanX (20).
